# Correction: Predicting wildlife corridors for multiple species in an East African ungulate community

**DOI:** 10.1371/journal.pone.0329292

**Published:** 2025-07-29

**Authors:** The PLOS One Staff

## Notice of Republication

An author’s name was misspelled. It has been updated from Danielle Radomille to Danielle Radomile.

## Supporting information

S1 File Originally published, uncorrected article. (PDF)

S2 File Republished, corrected article. (PDF)
