# Supplementary material for: Predicting wildlife corridors for multiple species in an East African ungulate community
Source: PLoS One. 2022 Apr 5;17(4):e0265136. doi: 10.1371/journal.pone.0265136 (PMC8982851; doi:10.1371/journal.pone.0265136)
Supplement: S1 File — contains all 12 supporting figures (S1-S12) referenced in the main text along with their associated captions. (DOCX) [file pone.0265136.s001.docx]

**Supplemental Figures for**

**“Predicting wildlife corridors for multiple species in an East African ungulate community”**


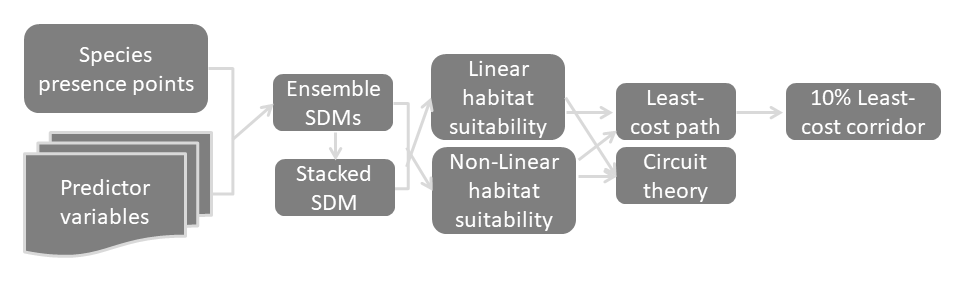


**Fig S1. Workflow indicating how species presence points and environmental predictor variables were used to build species distribution models, which in turn were used to model landscape connectivity.**


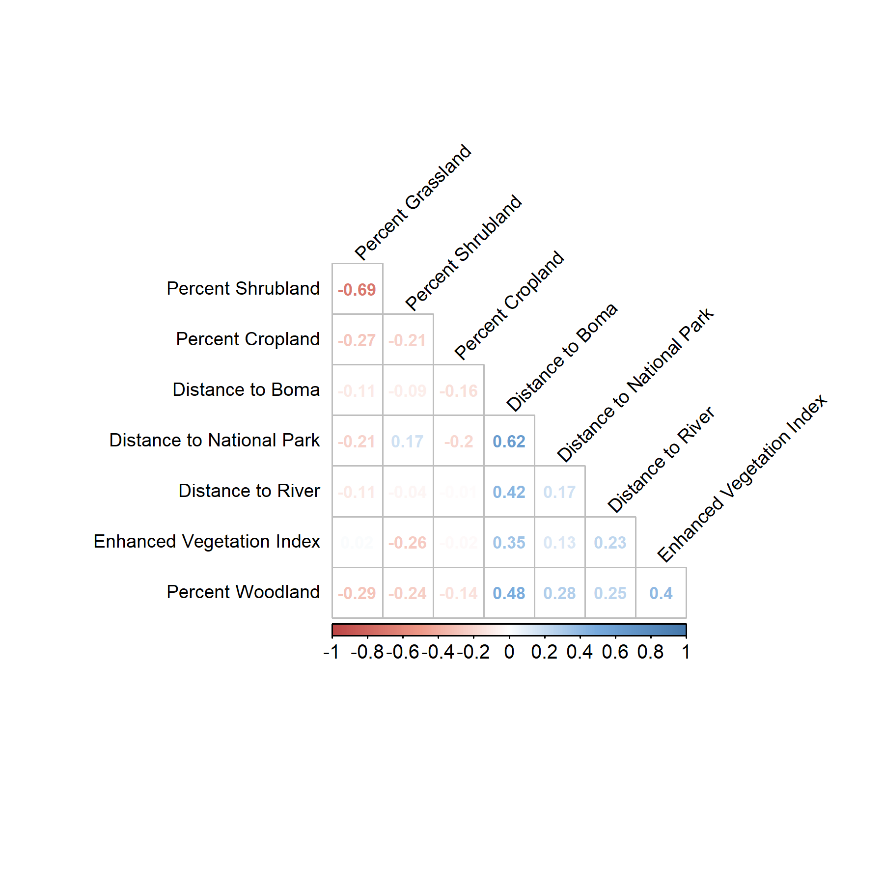


**Figure S2: Pearson correlation coefficients for the eight environmental predictor variables used to model large mammal habitat suitability visualized using the ‘corrplot’ package in R (Wei and Simko, 2017).**

**
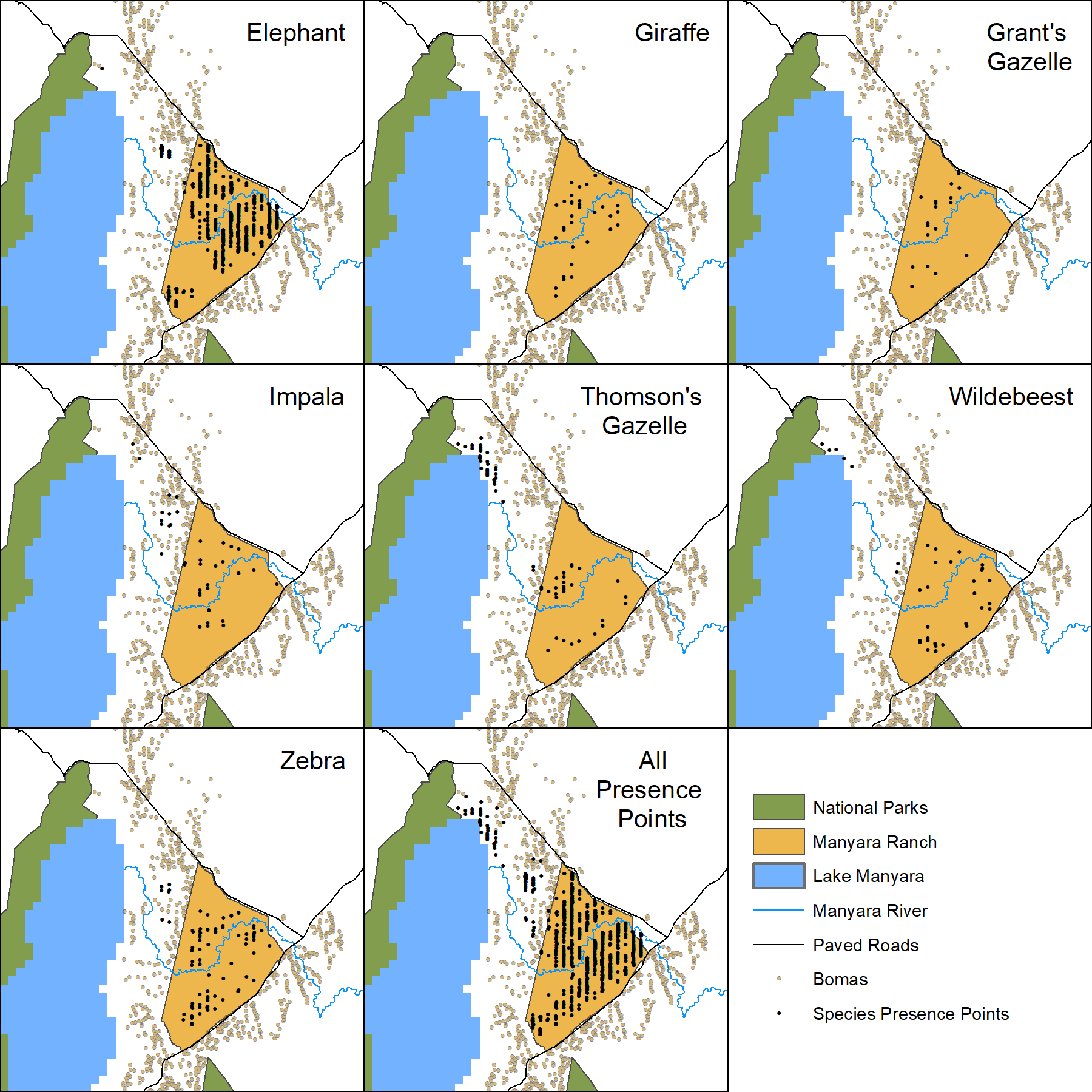
**

**Figure S3: Location of species presence points from transect surveys across the study area. Bomas are shown to illustrate the influence of human presence on species presence in the study area (Yamashita et al. 2018).**

**
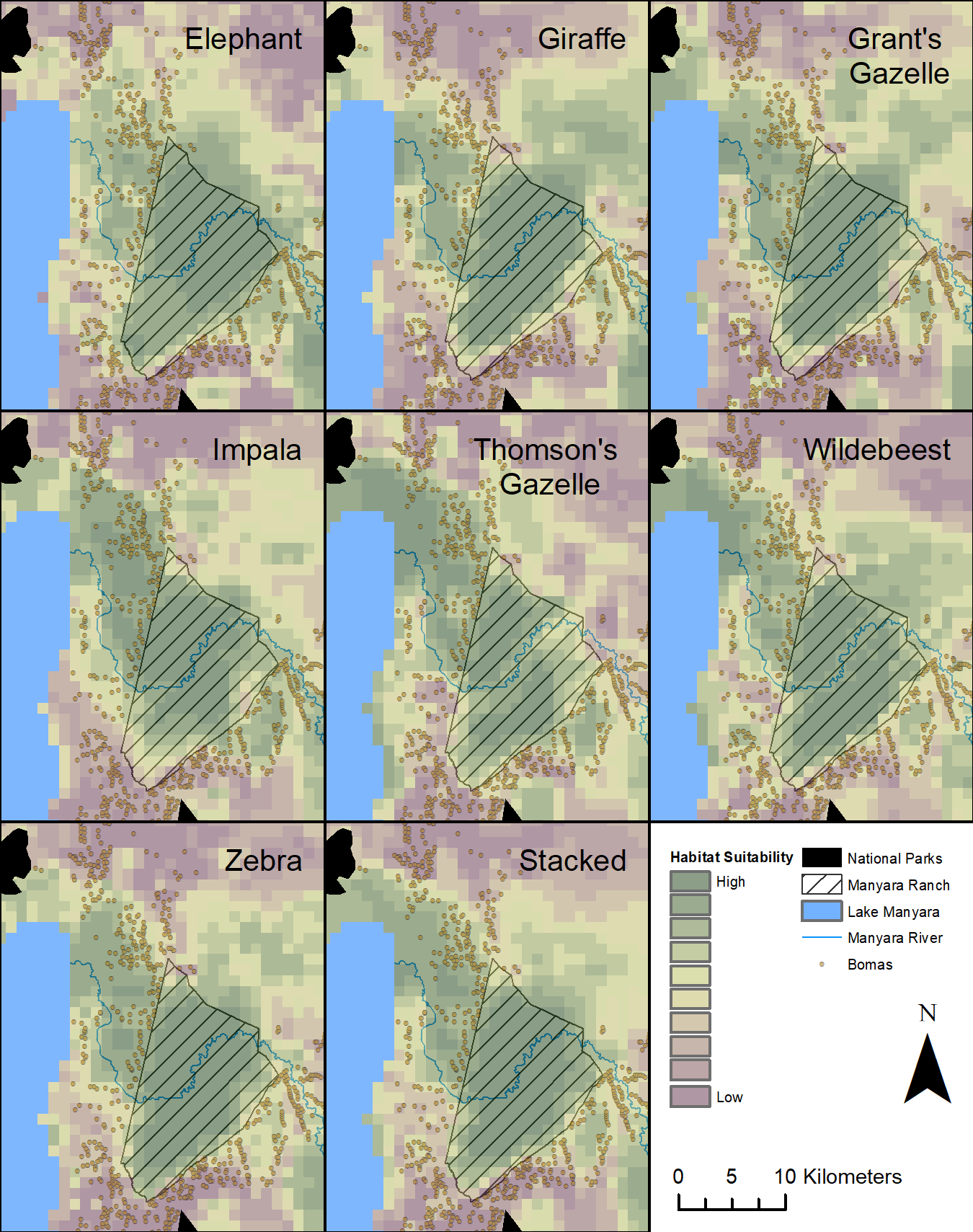
**

**Figure S4: Predicted habitat suitability for the linearly scaled single-species ensemble and stacked species distribution models. Bomas are shown to illustrate the influence of human presence on habitat suitability in the study area (Yamashita et al. 2018).**

**
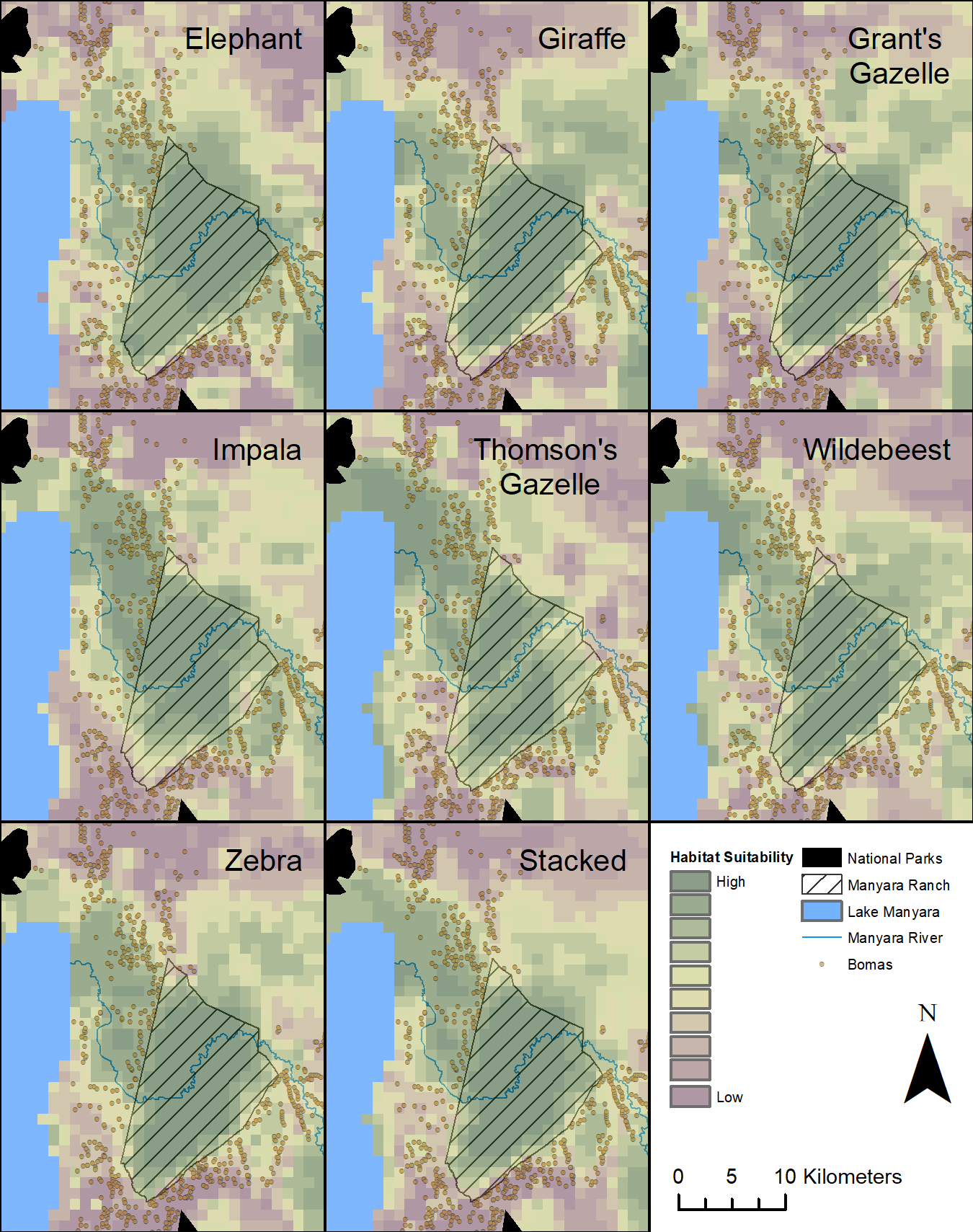
**

**Figure S5: Predicted habitat suitability for the non-linearly scaled single-species ensemble and stacked species distribution models. Bomas are shown to illustrate the influence of human presence on habitat suitability in the study area (Yamashita et al. 2018).**

**
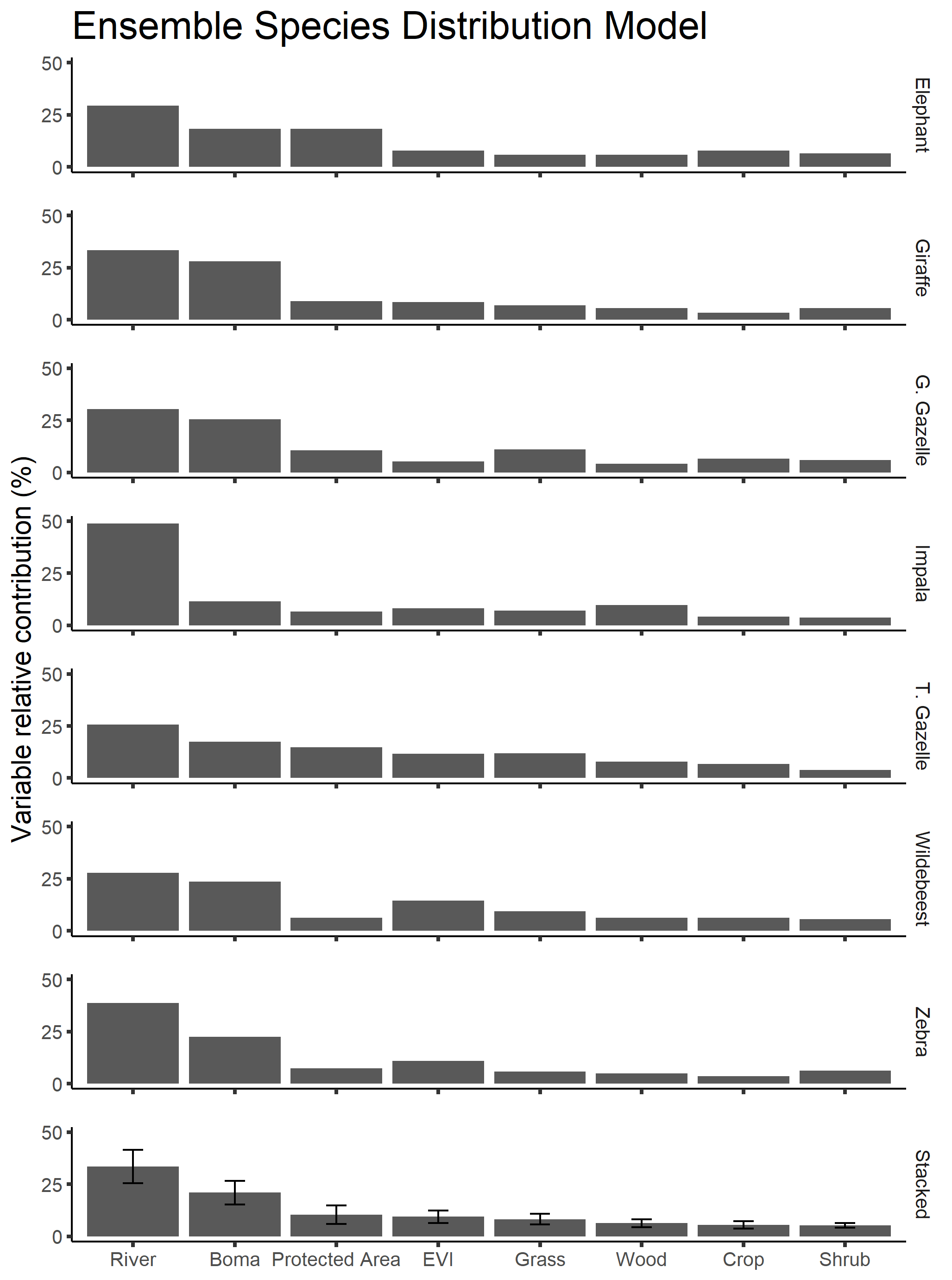
**

**Figure S6: Relative contributions of the eight environmental predictor variables to the single-species ensemble and stacked species distribution models.**


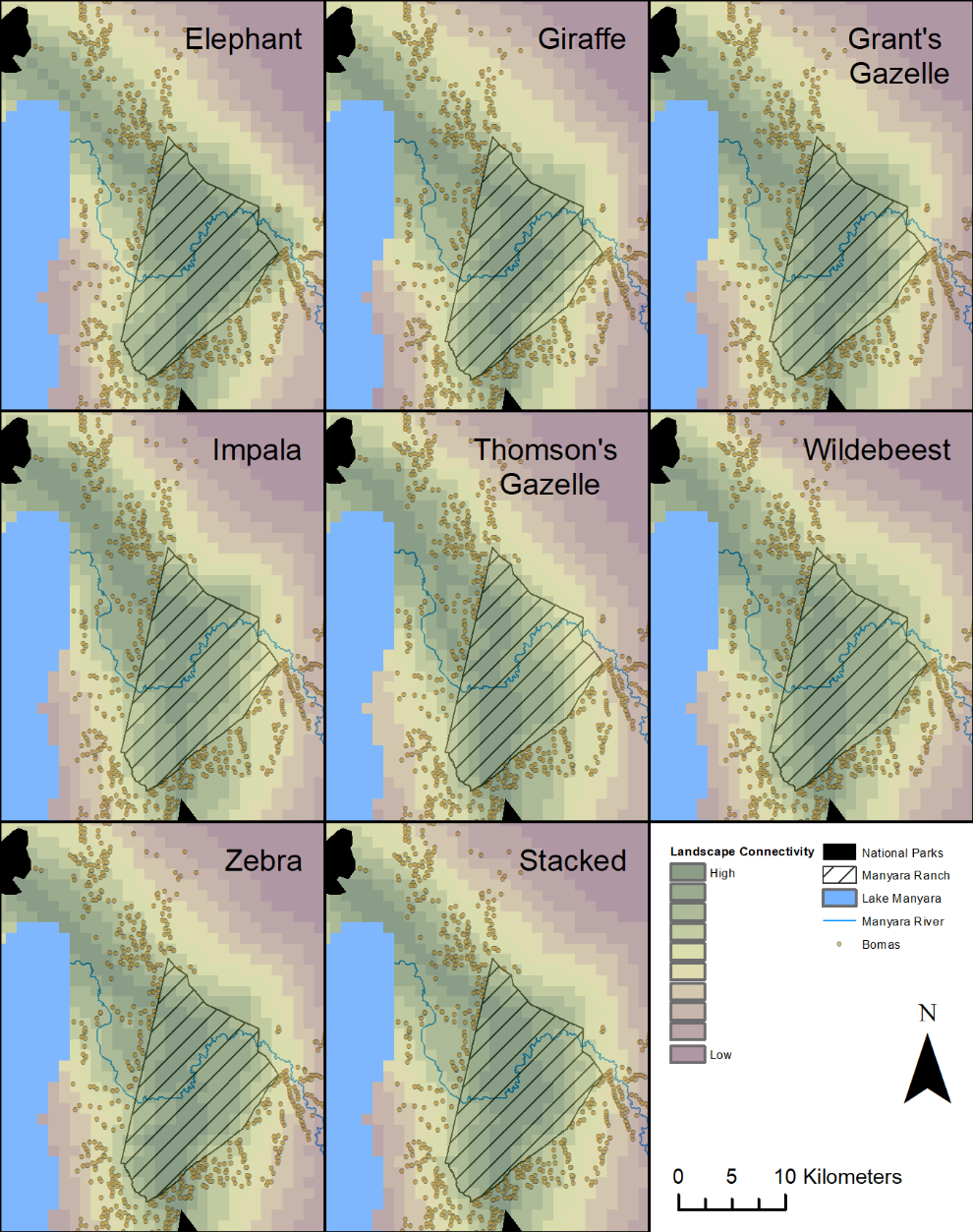


**Figure S7: Predicted landscape connectivity for the linearly scaled circuit theory single and stacked species models. Bomas are shown to illustrate the influence of human presence on predicted landscape connectivity in the study area (Yamashita et al. 2018).**


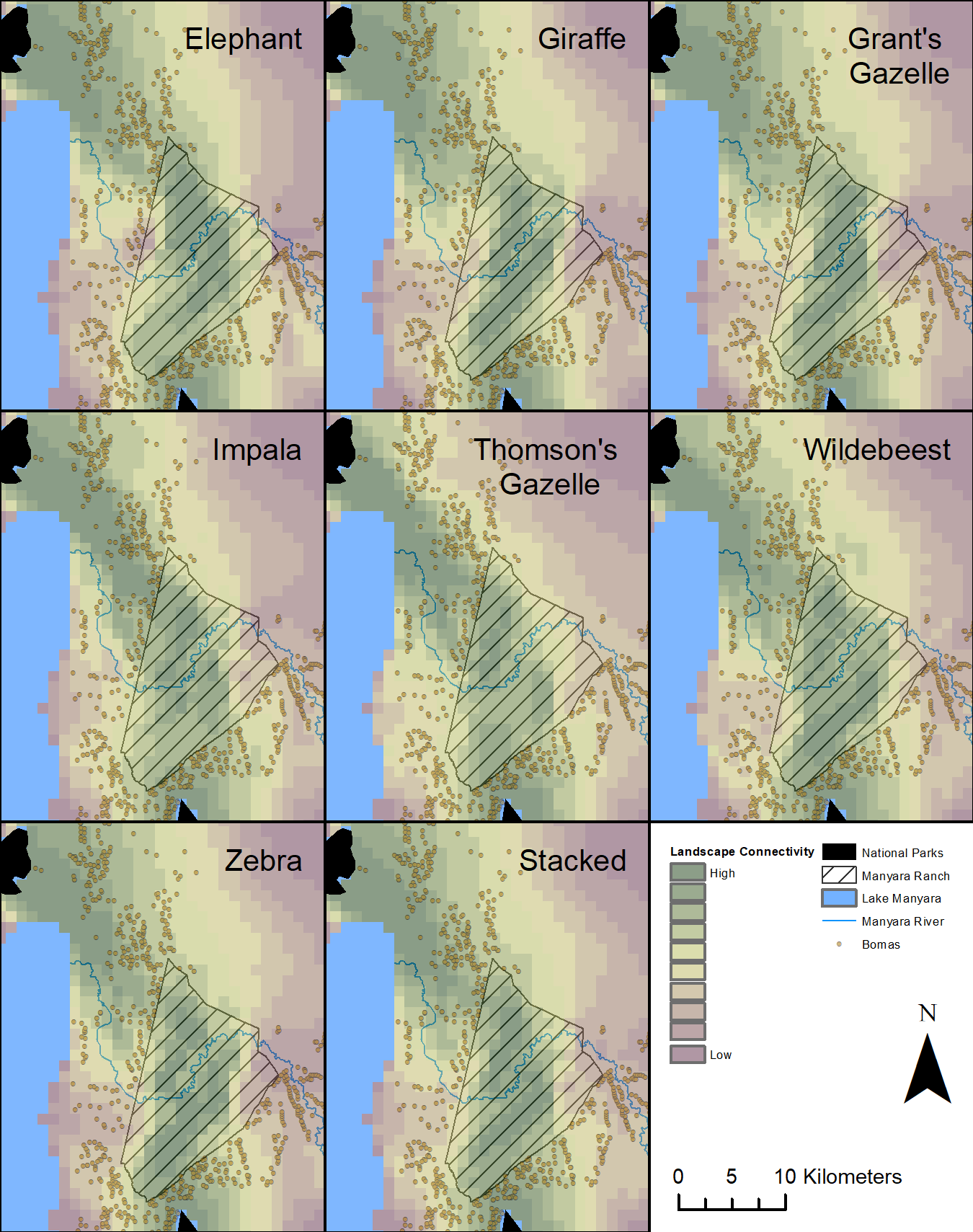


**Figure S8: Predicted landscape connectivity for the linearly scaled least-cost single and stacked species models. Bomas are shown to illustrate the influence of human presence on predicted landscape connectivity in the study area (Yamashita et al. 2018).**


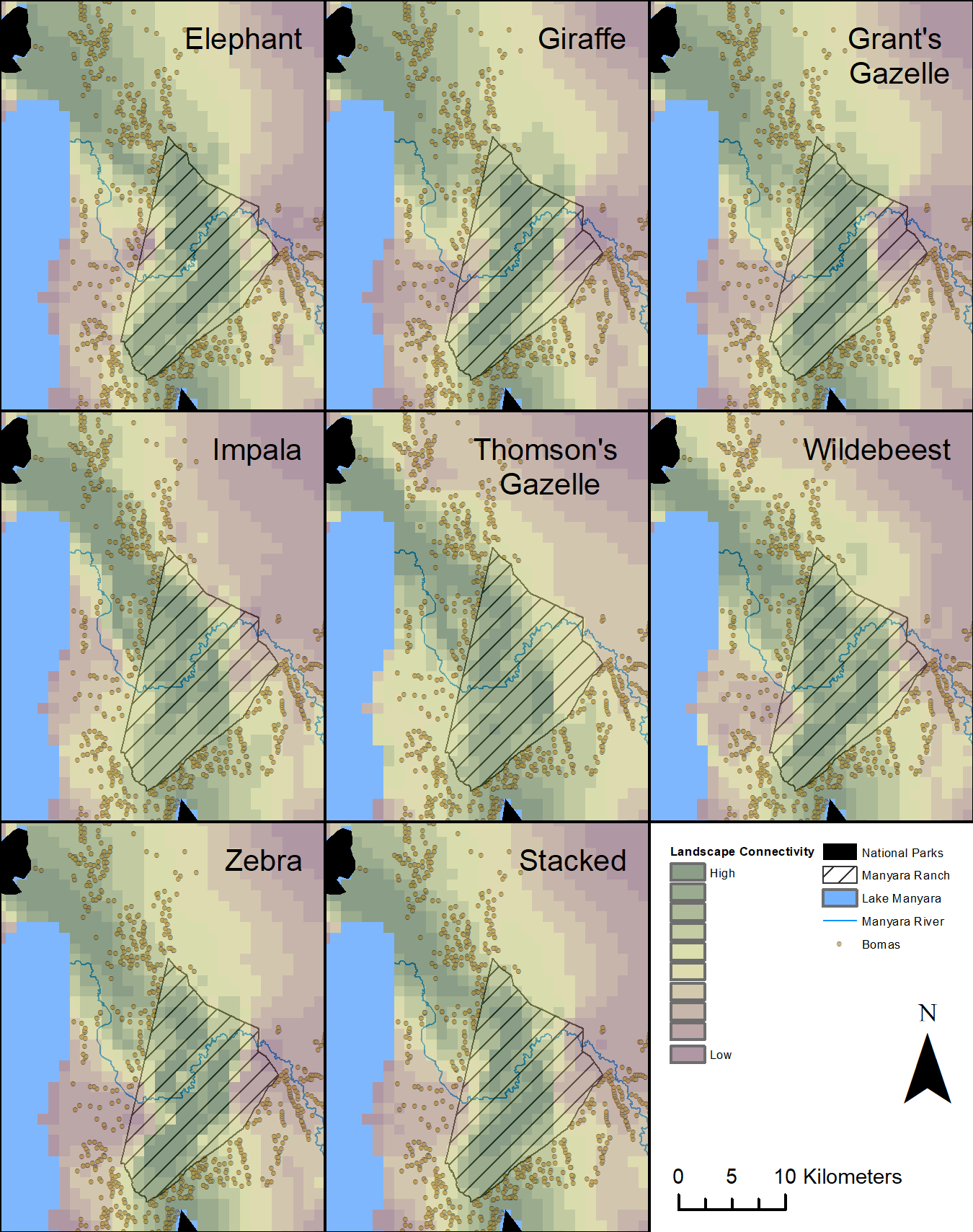


**Figure S9: Predicted landscape connectivity for the non-linearly scaled circuit theory single and stacked species models. Bomas are shown to illustrate the influence of human presence on predicted landscape connectivity in the study area (Yamashita et al. 2018).**


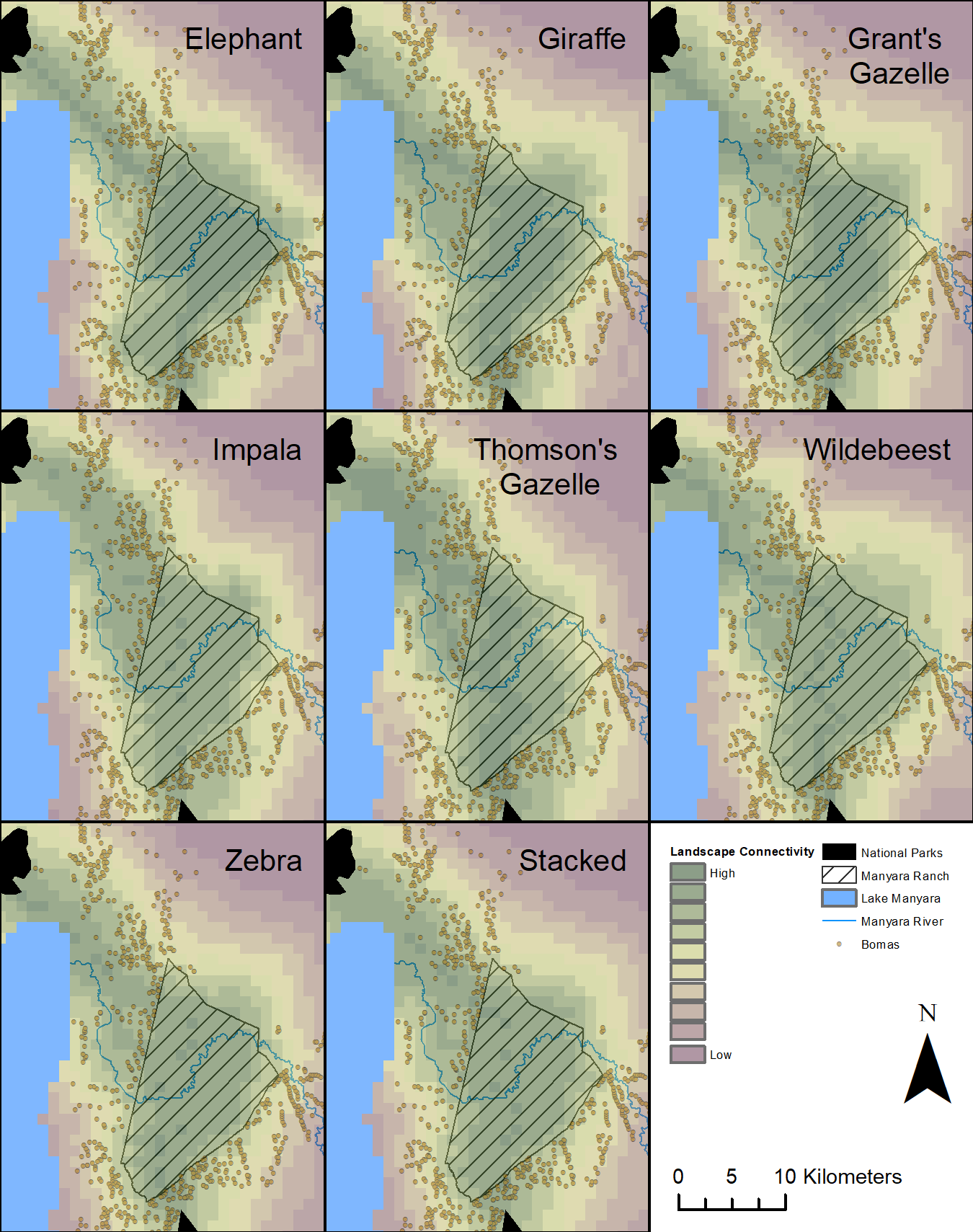


**Figure S10: Predicted landscape connectivity for the non-linearly scaled least-cost single and stacked species models. Bomas are shown to illustrate the influence of human presence on predicted landscape connectivity in the study area (Yamashita et al. 2018).**


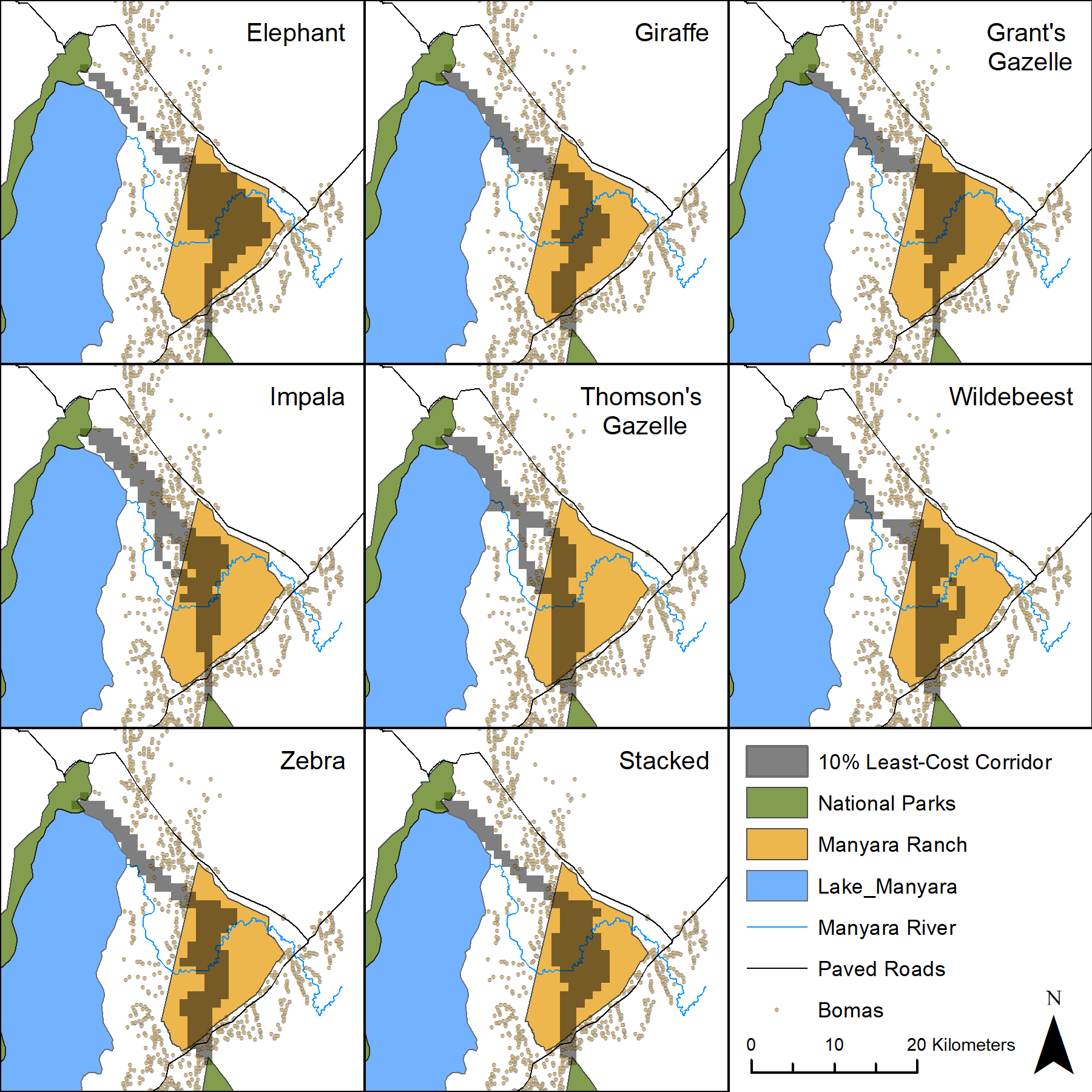


**Figure S11: Predicted 10% least-cost corridors across the study area for the linearly scaled single and stacked species models. Bomas are shown to illustrate the influence of human presence on predicted corridors in the study area (Yamashita et al. 2018).**


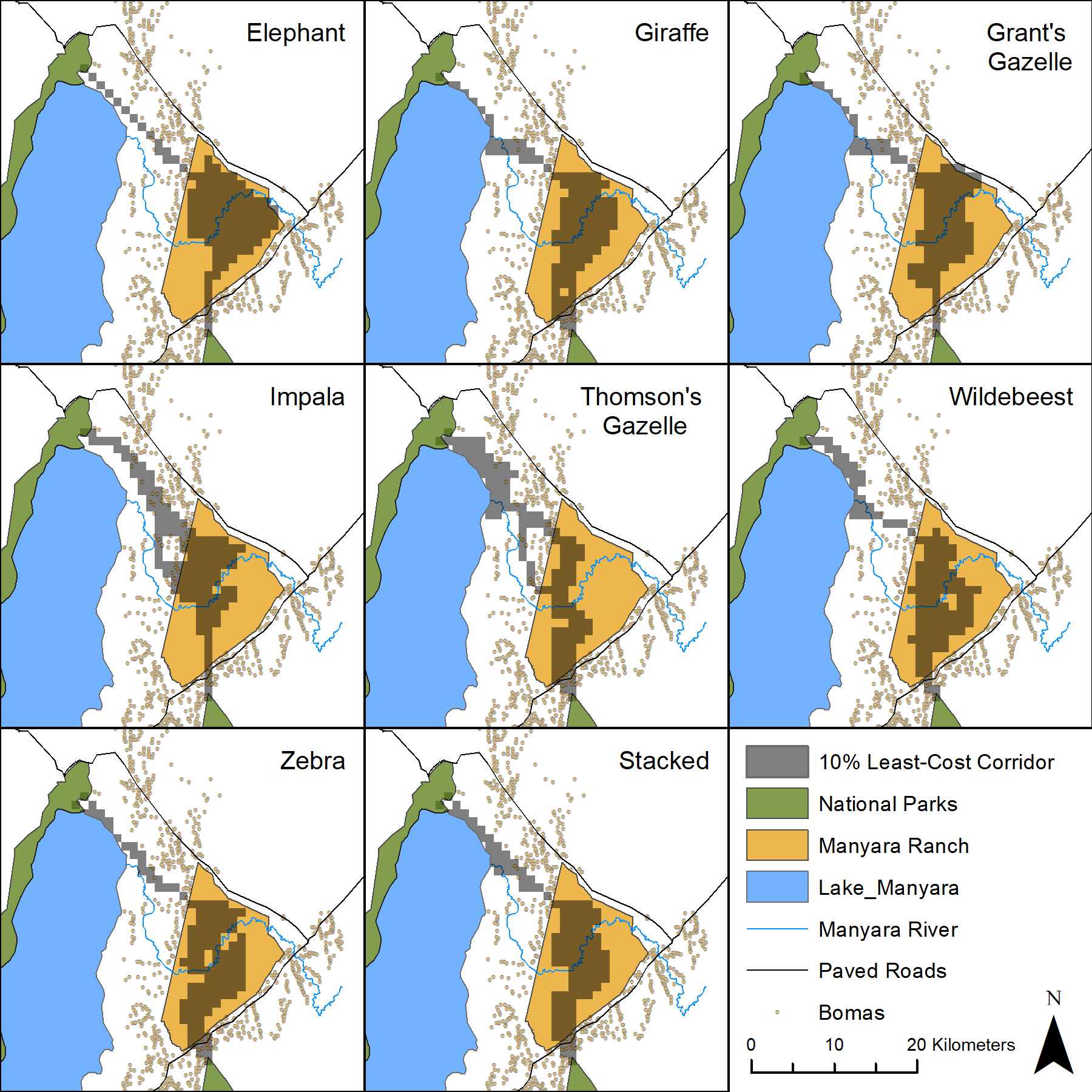


**Figure S12: Predicted 10% least-cost corridors across the study area for the non-linearly scaled single and stacked species models. Bomas are shown to illustrate the influence of human presence on predicted corridors in the study area (Yamashita et al. 2018).**
