# Supplementary material for: Correction: Predicting wildlife corridors for multiple species in an East African ungulate community
Source: PLoS One. 2025 Jul 29;20(7):e0329292. doi: 10.1371/journal.pone.0329292 (PMC12306748; doi:10.1371/journal.pone.0329292)
Supplement: S1 File — (PDF) [file pone.0329292.s001.pdf]

RESEARCH ARTICLE

Predicting wildlife corridors for multiple species in an East African ungulate community

Jason Riggio[1]*, **Katie Foreman**[2], **Ethan Freedman**[3], **Becky Gottlieb**[4], **David Hendler**[5], **Danielle Radomille**[6], **Ryan Rodriguez**[7], **Thomas Yamashita**[8], **John Kioko**[4], **Christian Kiffner**[4,9]

1 Department of Wildlife, Fish and Conservation Biology, Museum of Wildlife and Fish Biology, University of California, Davis, California, United States of America, **2** Department of Environmental Studies, Franklin and Marshall College, Lancaster, Pennsylvania, United States of America, **3** Department of Biology, Tufts University, Medford, Maryland, United States of America, **4** The School for Field Studies, Center for Wildlife Management Studies, Karatu, Tanzania, **5** Bard College, Annandale-on-Hudson, New York, United States of America, **6** Department of Geography and the Environment, Villanova University, Villanova, Pennsylvania, United States of America, **7** Department of Natural Resources, Cornell University, Ithaca, New York, United States of America, **8** Caesar Kleberg Wildlife Research Institute, Texas A&M University-Kingsville, Kingsville, Texas, United States of America, **9** Junior Research Group Human-Wildlife Conflict & Coexistence, Leibniz Centre for Agricultural Landscape Research (ZALF), Müncheberg, Germany

* jsriggio@ucdavis.edu

Check for updates

OPEN ACCESS

Citation: Riggio J, Foreman K, Freedman E, Gottlieb B, Hendler D, Radomille D, et al. (2022) Predicting wildlife corridors for multiple species in an East African ungulate community. PLoS ONE 17(4): e0265136. https://doi.org/10.1371/journal.pone.0265136

Editor: Frank H. Koch, USDA Forest Service, UNITED STATES

Received: July 30, 2021

Accepted: February 23, 2022

Published: April 5, 2022

Copyright: © 2022 Riggio et al. This is an open access article distributed under the terms of the Creative Commons Attribution License, which permits unrestricted use, distribution, and reproduction in any medium, provided the original author and source are credited.

Data Availability Statement: All relevant data are within the manuscript and its Supporting Information files.

Funding: The authors received no specific funding for this work.

Competing interests: The authors have declared that no competing interests exist.

Abstract

Wildlife corridors are typically designed for single species, yet holistic conservation approaches require corridors suitable for multiple species. Modelling habitat linkages for wildlife is based on several modelling steps (each involving multiple choices), and in the case of multi-species corridors, an approach to optimize single species corridors to few or a single functional corridor for multiple species. To model robust corridors for multiple species and simultaneously evaluate the impact of methodological choices, we develop a multi-method approach to delineate corridors that effectively capture movement of multiple wildlife species, while limiting the area required. Using wildlife presence data collected along ground-based line transects between Lake Manyara and Tarangire National Parks, Tanzania, we assessed species-habitat association in both ensemble and stacked species distribution frameworks and used these to estimate linearly and non-linearly scaled landscape resistances for seven ungulate species. We evaluated habitat suitability and least-cost and circuit theory-based connectivity models for each species individually and generated a multi-species corridor. Our results revealed that species-habitat relationships and subsequent corridors differed across species, but the pattern of predicted landscape connectivity across the study area was similar for all seven species regardless of method (circuit theory or least-cost) and scaling of the habitat suitability-based cost surface (linear or non-linear). Stacked species distribution models were highly correlated with the seven species for all model outputs (r = 0.79 to 0.97), while having the greatest overlap with the individual species least-cost corridors (linear model: 61.6%; non-linear model: 60.2%). Zebra was the best single-species proxy for landscape connectivity. Overall, we show that multi-species corridors based on stacked species distribution models achieve relatively low cumulative costs for

savanna ungulates as compared to their respective single-species corridors. Given the challenges and costs involved in acquiring data and parameterizing corridor models for multiple species, zebra may act as a suitable proxy species for ungulate corridor conservation in this system.

Introduction

East Africa features an impressive network of protected areas to safeguard landscapes, wildlife assemblages and associated ecosystem processes from human influences [1]. However, many of the protected areas are small, isolated, and not always effective in addressing these goals [2–5]. As a result, wildlife populations have been declining considerably across the region over the past decades [6–9]. One of the major concerns regarding the conservation of large mammal populations in Africa and elsewhere is the decline of functional connectivity within ecosystems [10–15]. Since connectivity is fundamental for effective landscape-scale conservation of wildlife populations [16], a key strategy to maintain or reverse the loss of functional connectivity is to identify and subsequently protect or restore wildlife corridors—i.e., patches of land that connect two or more protected areas or seasonal ranges of target species [11, 17, 18].

Most approaches to identify wildlife corridors in savanna ecosystems of Africa have focused on single species such as elephants (*Loxodonta africana*) [19–25], leopards (*Panthera pardus*) [26, 27], lions (*Panthera leo*) [28–30] or wildebeest (*Connochaetes taurinus*) [17, 31, 32]. Such a single species-focused approach is certainly justifiable if the target species (1) is of high conservation concern, (2) can be considered a keystone species, and (3) would maintain keystone processes in the ecosystem if protected [33–35]. However, if the aim is to protect multiple species, or a suite of ecological processes in a landscape that can only be maintained if several species can move across the landscape, conservation managers may be more inclined to identify and subsequently protect corridors that can effectively be used by multiple species [36–43].

Identification and delineation of multi-species corridors require spatially-explicit presence, genetic, or movement data for all target species, but such multi-species datasets are rarely available [44–46]. Beyond these logistical concerns, modelling multi-species corridors requires the assessment of cross-taxon trade-offs, as it is likely that a corridor designed for one species does not perfectly match corridor requirements for another species [38, 47, 48]. Failure to account for these cross-taxon differences could compromise the effectiveness of corridors as well as result in additional monetary costs if the primary focus were meant to preserve ideal corridors for all species [38]. Therefore, methods to delineate efficient multi-species corridors that will capture wildlife movement across a mammal community, while limiting the area required to protect them, could provide cost-effective options for connectivity conservation [49–51].

In tandem with the understanding of the biological and conservation importance of wildlife corridors, the theory and practice of corridor delineation has made substantial progress over the last two decades [52, 53]. Typically, corridor modelling involves (1) the collection of spatially-explicit animal distribution, genetic, or movement data, (2) assembling spatial variables that are hypothesized to be associated with animal presence or movement, (3) fitting appropriate habitat models which allow the prediction of how spatially explicit variables promote or impede animal movement or presence in the landscape, and (4) estimating corridors based on the spatial arrangement of the resistance surface [17, 54]. For all these steps, a variety of techniques and data conversion options are available (S1 Fig in S1 File). Because the choices made during each modelling step could have repercussions for the delineation of corridors,

comparing the relative effects of different model choices is a crucial step to illustrate uncertainty associated with corridor model parameterization [17, 45]. For example, to assess species-habitat associations, a variety of approaches and algorithms are available [55, 56]. To address this modelling uncertainty, we harnessed recent advances in species distribution modelling and used ensemble and stacked species distribution models to quantify species-habitat associations [57, 58]. Based on the species-habitat associations, scholars estimate resistance surfaces either by the inverse of the habitat suitability (assuming linear relationships between animal movement and landscape resistance) or based on non-linear transformation of the inverse of the squared habitat suitability (assuming non-linear relationships between animal movement and landscape resistance) [59]. As a final methodological choice, the use of either least-cost modelling or circuit theory (the two main approaches in corridor design) may also affect the design of corridors [60, 61]. The combined effects of these choices have rarely been quantified [62].

In this paper, we develop a multi-method approach to design corridors for a multi-species assemblage by combing habitat use data from seven ungulate species. Additionally, we assess the value of representative proxy species [63] as a cost-effective method to secure landscape permeability. By following a multi-method approach and quantifying the (relative) consequences of methodological choices for corridor design, we provide an analysis to illustrate uncertainty associated with our corridor modelling approach [45, 48]. We hypothesize that (1) species-habitat relationships (and thus movement costs) differ across species, (2) species-specific corridors vary in their spatial configuration, (3) multi-species corridors increase the movement costs for individual species but less so than other species-specific corridors, and (4) choices in the statistical methods and in parameterization of resistance surfaces affect corridor design.

Materials and methods

Study area

The study area is located centrally in the Tarangire-Manyara ecosystem, in a 1280 km^2 multi-use area between Lake Manyara (hereafter LMNP) and Tarangire (hereafter TNP) National Parks (centered approximately at 3.5846˚ S, 36.0021˚ E: Fig 2). The selected study area is ideal for testing multi-species corridor hypotheses because the landscape features a patchwork of protected areas [64] with large mammal populations still occupying land outside fully protected areas [65]. During the wet season, multiple wildlife species leave the national parks (primarily TNP; wildlife in LMNP do not exhibit regular, seasonal movements [2], but do occasionally move in and out of park boundaries [14, 66]) and move to areas that are often not formally protected [25, 66–70].

The study area is characterized by a semi-arid climate and the main vegetation type is *Acacia-Commiphora* savanna [71]. Human population growth and changes in traditional lifestyles have caused substantial expansion of human settlements and, consequently, conversion of natural vegetation to agriculture [72]. The western portion of the study area encompasses parts of the Mto wa Mbu Game Controlled Area, where wildlife falls under the jurisdiction of the Tanzanian Wildlife Authority. Settlements, agriculture, and livestock keeping are technically not permitted [73], yet enforcement is typically weak, and these land uses occur widely in the area [65]. The area also includes Manyara Ranch (hereafter MR), a multiple-use area designated to protect wildlife and support the pastoralist lifestyle of two adjacent villages (Esilalei and Oltukai). MR employs rangers to prevent hunting and ensure that livestock grazing regulations are followed. Livestock herds from adjacent villages are permitted to use the ranch during the dry season whereas ranch livestock graze the area year-round [65]. Settlements, agriculture, and

hunting are not permitted on the ranch. The small strip of land between MR and TNP is village lands in the Babati district (MR and Mto wa Mbu Game Controlled Area are situated in the Monduli district) and could not be sampled due to logistical constraints.

Wildlife transects

Wildlife presence data (elephant dung and sightings of Grant's gazelle [*Nanger granti*], giraffe [*Giraffa camelopardalis*], impala [*Aepyceros melampus*], Thomson's gazelle [*Eudorcas tomsonii*] wildebeest, and zebra [*Equus quagga*]) were collected along 248 one-km transects between LMNP and TNP (Fig 1). Each transect was surveyed once in April 2015, during the long rainy season. We recorded elephant dung instead of direct sightings as elephants traverse the human-dominated area but are rarely detected directly [74, 75]. The study area was divided into a 1 km by 1 km grid; transects bisected each grid north-south, such that parallel transects were separated by 1 km. Along each transect, we recorded sightings of all target species including the GPS coordinates and perpendicular distance from transect using a rangefinder.

All research was carried out with permission from the Tanzanian Wildlife Research Institute (TAWIRI) and the Tanzanian Commission for Science and Technology (COSTECH), permit #: 2014-324-ER-2013-191.

Habitat suitability models

To model large mammal species distributions, we created eight raster environmental predictor variables for the study area in ArcMap version 10.7.1 (ESRI, Redlands) (Fig 1). Variables were selected based on hypothesized, biological relationships to the presence of the target species [17]. We calculated the Euclidean distance from bomas (pastoral households and livestock enclosures), LMNP and TNP, and the Manyara River. The locations of bomas were digitized from Google Earth imagery that was captured between 2005 to 2013 [76]. We derived an Enhanced Vegetation Index (EVI) for the study area using the MODIS MOD13Q1 V6 Terra Vegetation Indices 16-Day Global 250 m dataset. Using Google Earth Engine, we took the median EVI value for each 250 m pixel in the study area for all six

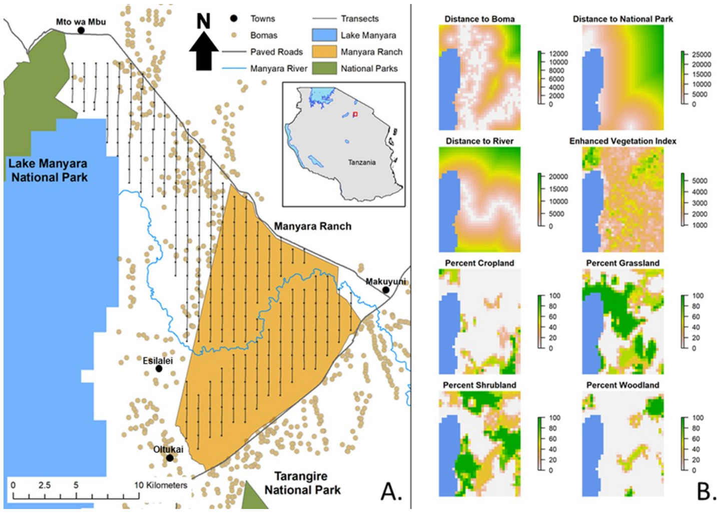

Fig 1. Maps of the study area and environmental predictor variables. (A.) Map of the study area in northern Tanzania (red box in inset map of Tanzania) showing the location of transects in relation to important landscape and management features including human settlements (bomas) [76]. (B.) Environmental predictor variables used to model large mammal habitat suitability. Distance variables are represented in meters.

https://doi.org/10.1371/journal.pone.0265136.g001

images captured during the 2015 long rainy season (image dates from April 6th to May 25th). The final four predictor variables were derived from the approximately 1 km resolution FAO Global Land Cover-SHARE database [77]. We used the percentage of density coverage layers for cropland, shrub covered area (shrubland) and tree covered area (woodland). For percentage of density coverage of grassland, we combined the grassland, herbaceous vegetation (aquatic or regularly flooded), and waterbodies layers. We masked out Lake Manyara from the study area using the Global Surface Water Occurrence dataset [78]. As the lake's water level fluctuates over the course of the year, we chose a threshold of 26% water occurrence, which represents the maximum threshold that could be applied without overlapping the transects walked in 2015. We projected all eight raster predictor layers to the Africa Albers Equal Area Conic coordinate system with a 1 km by 1 km cell size. None of the variables had a Pearson correlation coefficient greater than 0.7 (S2 Fig in S1 File), so all were retained for this analysis [79].

To predict species-specific habitat suitability across the study area, we built ensemble species distribution models for each species using the 'SSDM' package [57] in R version 4.0.3 [80]. We sampled environmental data from the eight predictor layers for the GPS locations of direct observations collected along the transects (i.e., presence points). Species with a sufficient sample size (i.e., $n > 13$) of direct observations [81] included giraffe (n = 34), Grant's gazelle (n = 19), impala (n = 37), Thomson's gazelle (n = 63), wildebeest (n = 36), and zebra (n = 85) (S3 Fig in S1 File). As transects resulted in few direct observations of elephants, we instead used GPS locations of elephant dung piles (n = 803). We performed geographic thinning to account for spatial biases [57], which reduced the number of elephant (n = 696) and Thomson's gazelle (n = 62) observations. Pseudo-absences were generated within the extent of the study area, with number and strategy dependent upon the type of model as recommended in [82]. Ensemble models were built from the highest performing models ($AUC \geq 0.90$) of eight different algorithms (generalized linear model, generalized additive model, multivariate adaptive regression splines, generalized boosted regressions model, classification tree analysis, random forest, maximum entropy, artificial neural network, and support vector machines). We evaluated the models using a 70% training/30% evaluation holdout method, with a total of 10 repetitions. To generate a stacked multi-species habitat suitability model, we summed the probabilities of the resulting habitat suitability maps.

Landscape connectivity models

To derive linearly scaled landscape resistance surfaces, we took the inverse of the species habitat suitability predictions from the ensemble models, multiplied them by 100 and added 1 [17, 61, 83]. This assumes that the cost-weighted distance of travelling across a cell with a predicted habitat suitability of 100% is 1 km (the distance to travel across the cell alone), while the cost-weighted distance of travelling through a cell with a predicted habitat suitability of 1% is equivalent to travelling 100 km through suitable habitat (or 100 times more difficult to cross than a cell with a predicted habitat suitability of 100%). In addition, we squared each of the resulting layers to generate non-linearly scaled cost surfaces [84] to address uncertainty in the relationship between habitat suitability and landscape resistance to movement. For each resulting cost surface layer (linear and non-linear), we modeled landscape connectivity between TNP and LMNP with circuit theory [85] and least-cost methods [86] using Linkage Mapper [87] in ArcMap version 10.7.1 (ESRI, Redlands). As a single cell-wide least-cost path is unlikely to represent wildlife movement, we mapped 10% least-cost corridors using the least-cost output [88, 89].

Comparing models

To compare how effective single species are at predicting habitat use and landscape connectivity for the other six species of ungulates in this study, we calculated pairwise Pearson correlations between the species-specific and stacked multi-species habitat suitability, circuit theory and least cost models. We also calculated the percent overlap between each of the single-species predicted least-cost corridor maps.

Results

Habitat suitability models

Ensemble model performance ranged from AUC = 0.91 (elephant) to AUC = 0.94 (Grant's gazelle) (S1 Table in S2 File). Predicted habitat suitability in the study area for all seven species was highest within MR for both the linearly and non-linearly scaled layers (Fig 2 and S4 and S5 Figs in S1 File). The northeastern corner of Lake Manyara was also predicted to be an area of relatively high habitat suitability. The top three variables with the greatest contribution to the models differed for each species (S6 Fig in S1 File and S2 Table in S2 File). Distance to the Manyara River was the predictor variable with the greatest contribution to the habitat suitability models for all species. Distance to bomas had the second highest overall contribution to the SSDM and was one of the top three predictor variables for all species. Distance to a national park had the third highest overall contribution to the SSDM.

Pairwise Pearson correlations between the species-specific and stacked multi-species habitat suitability models indicate a strong positive relationship (>0.7) between the predicted habitat suitability maps across the study area for all species except Thomson's gazelle for both

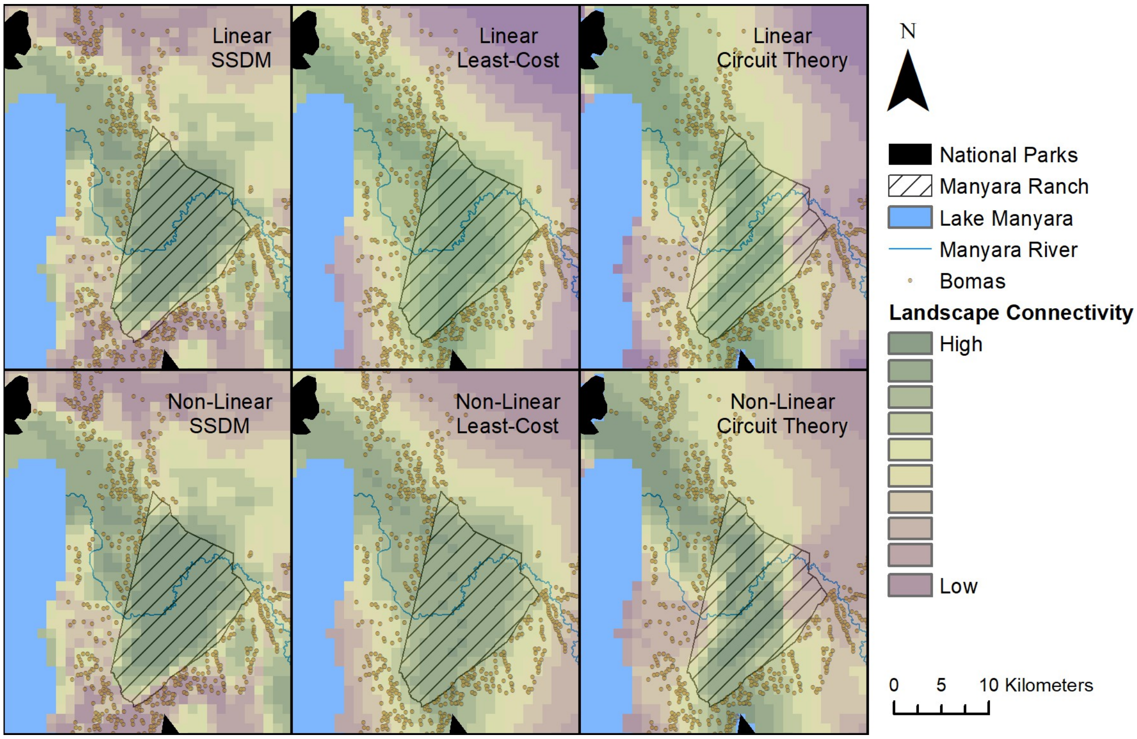

Fig 2. Predicted habitat suitability and least-cost and circuit theory-based landscape connectivity maps for the stacked species distribution model output in both linearly and non-linearly scaled layers. Bomas are shown to illustrate the influence of human presence on predicted habitat suitability in the study area [76].

https://doi.org/10.1371/journal.pone.0265136.g002

linearly and non-linearly scaled layers (Fig 3 and S4 Table in S2 File). The stacked multi-species model had the highest average pairwise correlation, followed by the zebra model (S3 Table in S2 File).

Landscape connectivity models

The pattern of predicted landscape connectivity across the study area between LMNP and TNP was similar for all seven species regardless of method (circuit theory or least-cost) and scaling of the habitat suitability-based cost surface (linear or non-linear) (Fig 2 and S7-S10 Figs in S1 File). Areas with the lowest landscape resistance ran from the northern tip of TNP across MR, before shifting northwest towards Lake Manyara.

Pairwise Pearson correlations between the species-specific and stacked multi-species landscape connectivity models indicated a strong positive relationship (>0.7) between the predicted circuit theory-derived connectivity maps across the study area for all species except Thomson's gazelle for non-linearly scaled layers (Fig 3 and S3 Table in S2 File). Similarly, the pairwise Pearson correlations showed strong positive relationships between the least-cost-derived connectivity maps, although the average correlation coefficient was >0.9 for all species and the multi-species model. The stacked multi-species model had the highest average pairwise correlation for both methods, followed by the zebra model (S3 Table in S2 File).

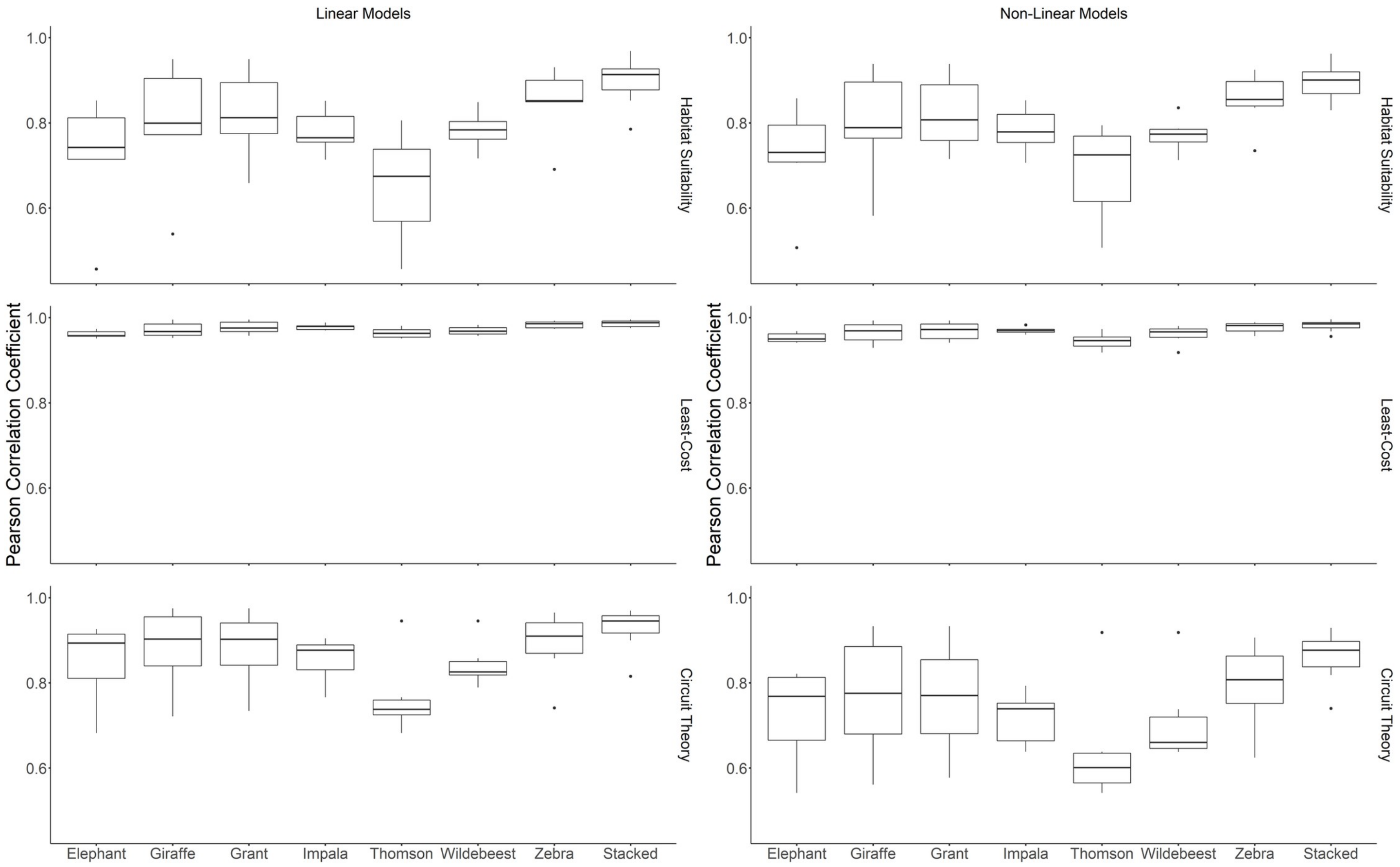

Fig 3. Average pairwise Pearson correlations between the species-specific and stacked multi-species linearly and non-linearly scaled landscape connectivity models.

https://doi.org/10.1371/journal.pone.0265136.g003

Least-cost corridor models

The landscape connectivity models predicted similar least-cost corridors for each of the seven focal species and the stacked multi-species model (Fig 4 and S11 and S12 Figs in S1 File). Travelling across the study area from Tarangire National Park in the south towards LMNP in the northwest (or vice versa), each species was predicted to move roughly directly across the unprotected lands between TNP and MR. Once in the ranch, each species was predicted to take slightly different optimal movement paths based on the species-specific habitat preferences. Predicted movement patterns appear to maximize the distance travelled through MR. Once exiting the ranch, each of the predicted least-cost corridors roughly converge in the grasslands at the northeastern edge of Lake Manyara and ultimately border the lake before entering LMNP (S11 and S12 Figs in S1 File). Similarly, the optimal multi-species least-cost corridor crosses directly from TNP into MR, maximizes the distance crossed within the ranch, and then directly crosses the Mto wa Mbu Game Controlled Area to reach the northeastern edge of Lake Manyara (Fig 4).

Average pairwise percent overlap between the least-cost corridor maps was generally high (>50%) for all species except elephant, impala (non-linearly scaled only), and Thomson's gazelle

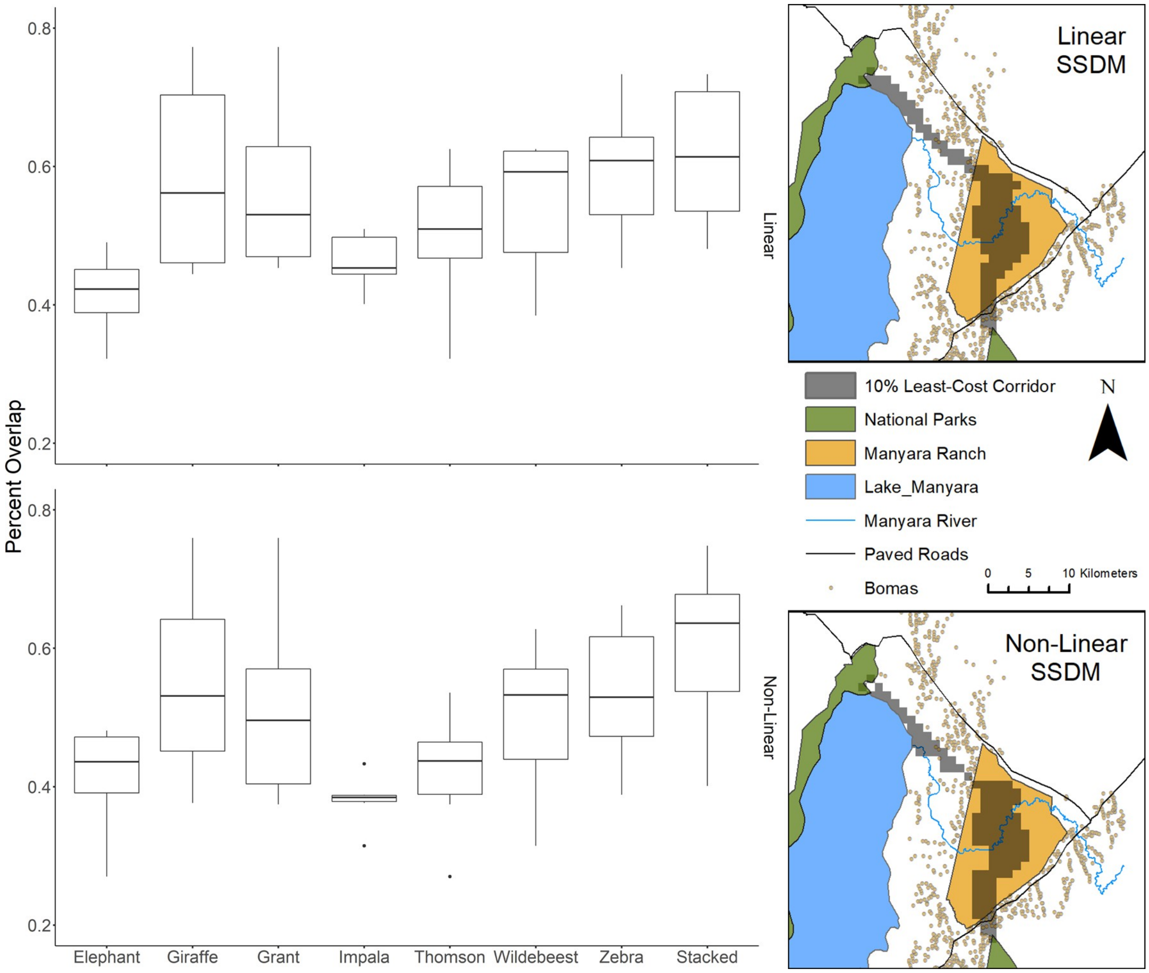

Fig 4. Average pairwise percent overlap between the species-specific and stacked multi-species linearly and non-linearly scaled 10% least-cost corridor maps. The predicted 10% least-cost corridors across the study area for the stacked species distribution models are shown on the right. Bomas are shown to illustrate the influence of human presence on predicted corridors [76].

https://doi.org/10.1371/journal.pone.0265136.g004

(non-linearly scaled only) (Fig 4). The multi-species stacked model had the highest average percent overlap for both the linearly and non-linearly scaled least-cost corridor maps (61.6% and 60.2% respectively), while zebra had the second highest average overlap for the linearly scaled model (59.4%) and giraffe for the non-linearly scaled model (55.0%) (S4 Table in S2 File).

Discussion

Multi-species corridors

Long-distance movement of vertebrates is a fundamental yet severely threatened process in terrestrial ecosystems across the world [90–92]. There is a growing recognition that wildlife corridors should be planned, designed, and implemented for multiple species so that, ideally, the full range of crucial ecosystem processes can be maintained across large spatial scales [36–43]. Conservation planning also ideally allows for movement of a variety of species to facilitate expected range shifts due to anticipated changes in climatic conditions [93–95]. Our results suggest that optimizing wildlife corridors by using either a multi-species or a proxy species approach can be a cost-effective method to secure landscape permeability for a large mammal assemblage. Using habitat suitability as a proxy for landscape resistance to movement for seven large mammal species suggests that a single multi-species wildlife corridor would best represent single-species landscape connectivity across the Tarangire-Manyara Ecosystem. However, gathering movement data for multiple species to derive an optimal multi-species corridor might be both time- and cost-prohibitive. Therefore, the identification of a single proxy species that most accurately captures landscape connectivity for an entire or a subset of a species assemblage could reduce these costs, particularly in areas where rapid land conversion is quickly threatening linkages between protected areas.

A previous study identified elephants as a proxy for other large mammal presence and movement in Tanzania [44]. In contrast, we found that zebra might better capture large mammal habitat use and movement in our study area. Differences in the ability of elephants and zebras to predict landscape use of a large mammal community in a typical Miombo ecosystem [44] and an *Acacia-Commiphora* dominated ecosystem (this study) may be related to actual or field method-related differences in mammal community composition in each ecosystem [47]. Irrespective of the underlying mechanism, the poor performance of elephants as a proxy for landscape connectivity in this study emphasizes that conservation planning by proxy species needs a site-specific evaluation to avoid suboptimal conservation outcomes [92]. Landscape connectivity models using both circuit theory and least-cost methods and linearly and non-linearly scaled habitat suitability surfaces for zebra had the highest correlation to connectivity models for the other six species. Furthermore, the percent overlap of the predicted least-cost corridor for zebra compared to the other six species was highest in the linearly scaled model and second highest for the non-linearly scaled model. These findings echo similar results that zebra presence is most closely associated with large mammal species richness in northern Tanzania, while elephants performed poorly as a proxy [47]. The ability of zebra to predict suitable corridors for a range of other herbivore species (including those that differ in feeding strategies) suggest that conservation efforts targeting the protection of corridors for zebra could help conserve landscape connectivity for large mammal assemblages in savanna Africa. Conceptually, space use of habitat generalists may best represent movement of a range of other species. Indeed, space use of zebra usually shows little response to spatial variables, which is indicative of a habitat generalist [96].

Considerations for corridor models

A key assumption in this study is that habitat suitability is analogous to, or at least a reasonable proxy for, landscape resistance. However, species distribution is not necessarily equivalent to

movement [54, 97]. The relationship between habitat suitability and movement has not been well studied in large mammal species in Africa, but in a recent case study, the distribution of wildebeest was found to be a suitable proxy for their movement [17]. Similarly, the distribution of elephants appears to be highly indicative of their movements as well [23]. However, landscape resistance may differ between day and nighttime [98, 99]. As the walking transects were conducted during daylight hours, our study does not capture the potential for differential nighttime habitat use when species might be moving across the landscape to avoid direct encounters with humans and livestock (although this caveat my not apply to elephant space use as the distribution of signs likely captures their actual distribution). Similarly, this study captures large mammal presence during one season of a single year. While the survey was conducted during the rainy season, when dispersal across this landscape generally occurs [66, 69, 100], spatio-temporal variation in resource availability (e.g., grass, non-alkaline surface water) and the level and extent of the alkaline Lake Manyara might alter landscape resistance. Therefore, optimal wildlife corridors in one year may offer suboptimal paths of movement in following years. However, this study does not primarily aim to offer guidance on where exactly to designate wildlife corridors in the Tarangire-Manyara ecosystem, and so the results from a single season and year are likely valid for testing how best to optimize wildlife corridors for multiple species. However, the consistent importance of distance from nearest boma as a predictor of wildlife presence (S6 Fig) indicates strong spatial avoidance of human settlements by all wildlife species in our study and highlights that human settlements add substantial movement costs to wildlife species [17].

Ideally, landscape resistance and corridor models can be cross-validated with movement data from the seven study species [23, 101]. Unfortunately, the only available movement data in the region is for wildebeest [100]. However, none of those five collared individuals entered LMNP, which prevents quantitative comparisons to our modeling efforts. Ideally, and given the importance of landscape connectivity for wildlife species in the fragmented Tarangire-Manyara ecosystem [67, 69, 70, 100, 102], additional efforts should be made to capture movement data for a greater number of species to inform corridor conservation in the region.

As we employed a suite of statistical methods and different parameterizations of the resistance surface, we were able to conduct an uncertainty analysis of our modelling choices. While often recommended, such analyses are rarely conducted [48]. Our approach to address uncertainty first considered species distribution model-derived habitat suitability maps as a proxy for landscape resistance. Many methods have been developed to model habitat suitability and rather than pick a single algorithm we chose instead to use an ensemble model in acknowledgment that while all models are flawed, each has their predictive strengths [103]. We then considered uncertainty in the relationship between habitat suitability and landscape resistance to movement. Using both linearly and non-linearly scaled resistance layers, we found that the resulting maps of connectivity were similar when comparing between species with the same species being more (e.g., zebra) or less (e.g., Thomson's gazelle) correlated to others regardless of the shape of transformation. Finally, we considered the impact of connectivity modeling method (least-cost versus circuit theory) on our multi-species comparisons. Similarly, we found that modeling method did not result in major differences in which species better predicted movement for the entire ungulate community.

Conclusions and conservation implications

To model robust corridors for multiple species and evaluate the impact of methodological choices, we developed a multi-method approach and parameterized corridor models for multiple species. Species-habitat relationships and subsequent corridors differed across species, but

the pattern of predicted landscape connectivity was similar for all seven species regardless of methodological choices. Stacked species distribution models were correlated with the seven species for all model outputs, while having the greatest overlap with the individual species least-cost corridors.

Connectivity in the fragmented Tarangire-Manyara ecosystem is rapidly declining [17, 67, 70]. To maintain functional connectivity and current wildlife population sizes (which are already likely below their historic baselines [2, 100]) and associated ecosystem services [104] conservation authorities need to implement effective and realistic wildlife corridors. Our field- and model-based results suggest that either a multi-species corridor or single species (zebra) corridor may effectively facilitate movement of the most abundant herbivore species in this ecosystem while minimizing the land that needs to be set aside for conservation. More generally, this approach highlights the potential feasibility of multi-species corridors for ensuring functional connectivity in savanna ecosystems and emphasizes the need for local evaluation of conservation by proxy approaches.

Supporting information

S1 File. Supporting figures. S1 File contains all 12 supporting figures (S1-S12) referenced in the main text along with their associated captions.
(DOCX)

S2 File. Supporting tables. S2 File contains all four supporting tables (S1-S4) referenced in the main text. Their associated captions follow: S1 Table. Ensemble species distribution model performance metrics. S2 Table. Relative contribution of environmental variables in explaining distribution of wildlife species between Tarangire and Lake Manyara National Parks, northern Tanzania. Some rows may not sum to precisely 100 due to rounding. S3 Table. Pearson correlation coefficients between the predicted habitat suitability maps derived by different methods for modelling the corridor (linear vs. non-linear scaled landscape resistances; least cost path modelling vs. circuit theory). S4 Table. Percent spatial overlap of wildlife corridors modelled with a least-cost algorithm and defining the resistance surface with either a linear or a non-linear scaled landscape resistance value.
(XLSX)

S1 Data. Supporting data. S1 Data contains the GPS locations of animal sightings used in these analyses.
(XLSX)

Acknowledgments

We sincerely thank the village authorities and the management of Manyara Ranch for granting access to the area. Fieldwork was greatly facilitated by the help of guides from the Mto wa Mbu Cultural Tourism Program and rangers from Manyara Ranch. We thank staff and management of the School for Field Studies for logistical support of this study.

Author Contributions

Conceptualization: Jason Riggio, John Kioko, Christian Kiffner.

Formal analysis: Jason Riggio, Christian Kiffner.

Investigation: Katie Foreman, Ethan Freedman, Becky Gottlieb, David Hendler, Danielle Radomille, Ryan Rodriguez, Thomas Yamashita, Christian Kiffner.

Writing – original draft: Jason Riggio, Christian Kiffner.

Writing – review & editing: Katie Foreman, Ethan Freedman, Becky Gottlieb, David Hendler, Danielle Radomille, Ryan Rodriguez, Thomas Yamashita, John Kioko.

References

1. Riggio J, Jacobson AP, Hijmans RJ, Caro T. How effective are the protected areas of East Africa? Global Ecology and Conservation. 2019; 17: e00573. https://doi.org/10.1016/j.gecco.2019.e00573
2. Kiffner C, Rheault H, Miller E, Scheetz T, Enriquez V, Swafford R, et al. Long-term population dynamics in a multi-species assemblage of large herbivores in East Africa. Ecosphere. 2017; 8: e02027. https://doi.org/10.1002/ecs2.2027
3. Mtui D, Owen-Smith N, Lepczyk C. Assessment of wildlife populations trends in three protected areas in Tanzania from 1991 to 2012. African Journal of Ecology. 2017; 55: 305–315. https://doi.org/10.1111/aje.12354
4. Newmark WD. Isolation of African protected areas. Frontiers in Ecology and the Environment. 2008; 6: 321–328. https://doi.org/10.1890/070003
5. Newmark WD. Insularization of Tanzanian parks and the local extinction of large mammals. Conservation Biology. 1996; 10: 1549–1556. https://doi.org/10.1046/j.1523-1739.1996.10061549.x
6. Craigie ID, Baillie JEM, Balmford A, Carbone C, Collen B, Green RE, et al. Large mammal population declines in Africa's protected areas. Biological Conservation. 2010; 143: 2221–2228. https://doi.org/10.1016/J.BIOCON.2010.06.007
7. Ogutu JO, Piepho HP, Said MY, Ojwang GO, Njino LW, Kifugo SC, et al. Extreme wildlife declines and concurrent increase in livestock numbers in Kenya: What are the causes? Paiva SR, editor. PLoS ONE. 2016; 11: e0163249. https://doi.org/10.1371/journal.pone.0163249 PMID: 27676077
8. Stoner C, Caro T, Mduma S, Mlingwa C, Sabuni G, Borner M, et al. Changes in large herbivore populations across large areas of Tanzania. African Journal of Ecology. 2007; 45: 202–215. https://doi.org/10.1111/j.1523-1739.2007.00705.x PMID: 17531042
9. Western D, Russell S, Cuthil I. The status of wildlife in protected areas compared to non-protected areas of Kenya. Somers M, editor. PLoS ONE. 2009; 4: e6140. https://doi.org/10.1371/journal.pone.0006140 PMID: 19584912
10. Beale CM, Rensberg S van, Bond WJ, Coughenour M, Fynn RWS, Gaylard A, et al. Ten lessons for the conservation of African savannah ecosystems. Biological Conservation. 2013; 167: 224–232. https://doi.org/10.1016/j.biocon.2013.08.025
11. Caro T, Jones T, Davenport TRB. Realities of documenting wildlife corridors in tropical countries. Biological Conservation. 2009; 142: 2807–2811. https://doi.org/10.1016/j.biocon.2009.06.011
12. Fynn RWS, Bonyongo MC. Functional conservation areas and the future of Africa's wildlife. African Journal of Ecology. 2011; 49: 175–188. https://doi.org/10.1111/j.1365-2028.2010.01245.x
13. Riggio J, Caro T. Structural connectivity at a national scale: Wildlife corridors in Tanzania. Lepczyk CA, editor. PLoS ONE. 2017; 12: e0187407. https://doi.org/10.1371/journal.pone.0187407 PMID: 29095901
14. Lee DE, Bolger DT. Movements and source–sink dynamics of a Masai giraffe metapopulation. Population Ecology. 2017; 59: 157–168. https://doi.org/10.1007/s10144-017-0580-7
15. Wegmann M, Santini L, Leutner B, Safi K, Rocchini D, Bevanda M, et al. Role of African protected areas in maintaining connectivity for large mammals. Philosophical Transactions of the Royal Society B: Biological Sciences. 2014;369. https://doi.org/10.1098/rstb.2013.0193 PMID: 24733948
16. Hansen AJ, DeFries R. Ecological mechanisms linking protected areas to surrounding lands. Ecological Applications. 2007; 17: 974–988. https://doi.org/10.1890/05-1098 PMID: 17555212
17. Bond ML, Bradley CM, Kiffner C, Morrison TA, Lee DE. A multi-method approach to delineate and validate migratory corridors. Landscape Ecology. 2017; 32: 1705–1721. https://doi.org/10.1007/s10980-017-0537-4
18. Jones T, Bamford AJ, Ferrol-Schulte D, Hieronimo P, Mcwilliam N, Rovero F. Vanishing wildlife corridors and options for restoration: A case study from Tanzania. Tropical Conservation Science. 2012; 5: 463–474. https://doi.org/10.1177/194008291200500405
19. Roever CL, van Aarde RJ, Leggett K. Functional connectivity within conservation networks: Delineating corridors for African elephants. Biological Conservation. 2013; 157: 128–135. https://doi.org/10.1016/j.biocon.2012.06.025

20. Kikoti AP, Griffin CR, Pamphii L. Elephant use and conflict leads to Tanzania's first wildlife conservation corridor. Pachyderm. 2010; 48: 57–66.

21. van de Perre F, Adriaensen F, Songorwa AN, Leirs H. Locating elephant corridors between Saadani National Park and the Wami-Mbiki Wildlife Management Area, Tanzania. African Journal of Ecology. 2014; 52: 448–457. https://doi.org/10.1111/aje.12139

22. Schüßler D, Lee PC, Stadtmann R. Analyzing land use change to identify migration corridors of African elephants (Loxodonta africana) in the Kenyan-Tanzanian borderlands. Landscape Ecology. 2018; 33: 2121–2136. https://doi.org/10.1007/s10980-018-0728-7

23. Osipova L, Okello MM, Njumbi SJ, Ngene S, Western D, Hayward MW, et al. Validating movement corridors for African elephants predicted from resistance-based landscape connectivity models. Landscape Ecology. 2019; 34: 865–878. https://doi.org/10.1007/s10980-019-00811-0

24. Cisneros-Araujo P, Ramirez-Lopez M, Juffe-Bignoli D, Fensholt R, Muro J, Mateo-Sánchez MC, et al. Remote sensing of wildlife connectivity networks and priority locations for conservation in the Southern Agricultural Growth Corridor (SAGCOT) in Tanzania. Pettorelli N, Buchanan G, editors. Remote Sensing in Ecology and Conservation. 2021; rse2.199. https://doi.org/10.1002/rse2.171 PMID: 33889421

25. Pittiglio C, Skidmore AK, van Gils HAMJ, Prins HHT. Identifying transit corridors for elephant using a long time-series. International Journal of Applied Earth Observation and Geoinformation. 2012; 14: 61–72. https://doi.org/10.1016/j.jag.2011.08.006

26. Pitman RT, Fattebert J, Williams ST, Williams KS, Hill RA, Hunter LTB, et al. Cats, connectivity and conservation: incorporating data sets and integrating scales for wildlife management. Howe C, editor. Journal of Applied Ecology. 2017; 54: 1687–1698. https://doi.org/10.1111/1365-2664.12851

27. Fattebert J, Robinson HS, Balme G, Slotow R, Hunter L. Structural habitat predicts functional dispersal habitat of a large carnivore: How leopards change spots. Ecological Applications. 2015; 25: 1911–1921. https://doi.org/10.1890/14-1631.1 PMID: 26591456

28. Cushman SA, Elliot NB, Macdonald DW, Loveridge AJ. A multi-scale assessment of population connectivity in African lions (Panthera leo) in response to landscape change. Landscape Ecology. 2016; 31: 1337–1353. https://doi.org/10.1007/s10980-015-0292-3

29. Cushman SA, Elliot NB, Bauer D, Kesch K, Bahaa-el-din L, Bothwell H, et al. Prioritizing core areas, corridors and conflict hotspots for lion conservation in southern Africa. Cameron EZ, editor. PLoS ONE. 2018; 13: e0196213. https://doi.org/10.1371/journal.pone.0196213 PMID: 29975694

30. Elliot NB, Cushman SA, Macdonald DW, Loveridge AJ. The devil is in the dispersers: Predictions of landscape connectivity change with demography. Pettorelli N, editor. Journal of Applied Ecology. 2014; 51: 1169–1178. https://doi.org/10.1111/1365-2664.12282

31. Morrison TA, Bolger DT. Connectivity and bottlenecks in a migratory wildebeest Connochaetes taurinus population. Oryx. 2014; 48: 613–621. https://doi.org/10.1017/s0030605313000537

32. Thirgood S, Mosser A, Tham S, Hopcraft G, Mwangomo E, Mlengeya T, et al. Can parks protect migratory ungulates? The case of the Serengeti wildebeest. Animal Conservation. 2004; 7: 113–120. https://doi.org/10.1017/S1367943004001404

33. Caro T, O'Doherty G. On the use of surrogate species in conservation biology. Conservation Biology. 1999; 13: 805–814. https://doi.org/10.1046/j.1523-1739.1999.98338.x

34. Caro T. Umbrella species: Critique and lessons from East Africa. Animal Conservation. 2003; 6: 171–181. https://doi.org/10.1017/S1367943003003214

35. Sinclair ARE. Mammal population regulation, keystone processes and ecosystem dynamics. Philosophical Transactions of the Royal Society B: Biological Sciences. 2003; 358: 1729–1740. https://doi.org/10.1098/rstb.2003.1359 PMID: 14561329

36. Brodie JF, Giordano AJ, Dickson BG, Hebblewhite M, Bernard H, Mohd-Azlan J, et al. Evaluating multispecies landscape connectivity in a threatened tropical mammal community. Conservation Biology. 2015; 29: 122–132. https://doi.org/10.1111/cobi.12337 PMID: 25065425

37. DeMatteo KE, Rinas MA, Zurano JP, Selleski N, Schneider RG, Argüelles CF. Using niche-modelling and species-specific cost analyses to determine a multispecies corridor in a fragmented landscape. Heneberg P, editor. PLoS ONE. 2017; 12: e0183648. https://doi.org/10.1371/journal.pone.0183648 PMID: 28841692

38. Dilkina B, Houtman R, Gomes CP, Montgomery CA, McKelvey KS, Kendall K, et al. Trade-offs and efficiencies in optimal budget-constrained multispecies corridor networks. Conservation Biology. 2017; 31: 192–202. https://doi.org/10.1111/cobi.12814 PMID: 27677418

39. Koen EL, Bowman J, Sadowski C, Walpole AA. Landscape connectivity for wildlife: Development and validation of multispecies linkage maps. Tatem A, editor. Methods in Ecology and Evolution. 2014; 5: 626–633. https://doi.org/10.1111/2041-210X.12197

40. Liu C, Newell G, White M, Bennett AF. Identifying wildlife corridors for the restoration of regional habitat connectivity: A multispecies approach and comparison of resistance surfaces. Yue B-S, editor. PLoS ONE. 2018; 13: e0206071. https://doi.org/10.1371/journal.pone.0206071 PMID: 30403713
41. Marrotte RR, Bowman J, Brown MGC, Cordes C, Morris KY, Prentice MB, et al. Multi-species genetic connectivity in a terrestrial habitat network. Movement Ecology. 2017; 5: 21. https://doi.org/10.1186/s40462-017-0112-2 PMID: 29043084
42. Khosravi R, Hemami MR, Cushman SA. Multispecies assessment of core areas and connectivity of desert carnivores in central Iran. Thuiller W, editor. Diversity and Distributions. 2018; 24: 193–207. https://doi.org/10.1111/ddi.12672
43. Crego RD, Wells HBM, Ndung'u KS, Evans L, Njeri Nduguta R, Chege MA, et al. Moving through the mosaic: identifying critical linkage zones for large herbivores across a multiple-use African landscape. Landscape Ecology. 2021; 1–16. https://doi.org/10.1007/s10980-021-01232-8
44. Epps CW, Mutayoba BM, Gwin L, Brashares JS. An empirical evaluation of the African elephant as a focal species for connectivity planning in East Africa. Diversity and Distributions. 2011; 17: 603–612. https://doi.org/10.1111/j.1472-4642.2011.00773.x
45. Beier P, Majka DR, Spencer WD. Forks in the road: Choices in procedures for designing wildland linkages. Conservation Biology. 2008; 22: 836–851. https://doi.org/10.1111/j.1523-1739.2008.00942.x PMID: 18544090
46. Brennan A, Beytell P, Aschenborn O, du Preez P, Funston PJ, Hanssen L, et al. Characterizing multispecies connectivity across a transfrontier conservation landscape. Journal of Applied Ecology. 2020; 57: 1700–1710. https://doi.org/10.1111/1365-2664.13716
47. Kiffner C, Albertini M, Ede A, Donnellan B, Hahn N, McGinnis MA, et al. Performance of individual species as indicators for large mammal species richness in Northern Tanzania. Ecological Indicators. 2015; 53: 70–77. https://doi.org/10.1016/j.ecolind.2015.01.022
48. Beier P, Majka DR, Newell SL. Uncertainty analysis of least-cost modeling for designing wildlife linkages. Ecological Applications. 2009; 19: 2067–2077. https://doi.org/10.1890/08-1898.1 PMID: 20014579
49. Meyer NFV, Moreno R, Reyna-Hurtado R, Signer J, Balkenhol N. Towards the restoration of the Mesoamerican Biological Corridor for large mammals in Panama: Comparing multi-species occupancy to movement models. Movement Ecology. 2020; 8: 3. https://doi.org/10.1186/s40462-019-0186-0 PMID: 31938545
50. Pliscoff P, Simonetti JA, Grez AA, Vergara PM, Barahona-Segovia RM. Defining corridors for movement of multiple species in a forest-plantation landscape. Global Ecology and Conservation. 2020; 23: e01108. https://doi.org/10.1016/j.gecco.2020.e01108
51. Mimet A, Clauzel C, Foltête JC. Locating wildlife crossings for multispecies connectivity across linear infrastructures. Landscape Ecology. 2016; 31: 1955–1973. https://doi.org/10.1007/s10980-016-0373-y
52. Aune K, Beier P, Hilty J, Shilling F. Assessment and Planning for Ecological Connectivity: A Practical Guide. Bozeman, MT., USA: Wildlife Conservation Society; 2011.
53. Cushman SA, McRae BH, Adriaensen F, Beier P, Shirley M, Zeller KA. Biological corridors and connectivity. Key Topics in Conservation Biology 2. 2013. pp. 384–404. https://doi.org/10.1002/9781118520178.ch21
54. Zeller KA, McGarigal K, Whiteley AR. Estimating landscape resistance to movement: A review. Landscape Ecology. 2012; 27: 777–797. https://doi.org/10.1007/s10980-012-9737-0
55. Bellamy C, Boughey K, Hawkins C, Reveley S, Spake R, Williams C, et al. A sequential multi-level framework to improve habitat suitability modelling. Landscape Ecology 2020 35:4. 2020;35: 1001–1020. https://doi.org/10.1007/S10980-020-00987-W
56. Smeraldo S, Bosso L, Salinas-Ramos VB, Ancillotto L, Sánchez-Cordero V, Gazaryan S, et al. Generalists yet different: distributional responses to climate change may vary in opportunistic bat species sharing similar ecological traits. Mammal Review. 2021; 51: 571–584. https://doi.org/10.1111/MAM.12247
57. Schmitt S, Pouteau R, Justeau D, de Boissieu F, Birnbaum P. ssdm: An r package to predict distribution of species richness and composition based on stacked species distribution models. Golding N, editor. Methods in Ecology and Evolution. 2017; 8: 1795–1803. https://doi.org/10.1111/2041-210X.12841
58. Hao T, Elith J, Guillera-Arroita G, Lahoz-Monfort JJ. A review of evidence about use and performance of species distribution modelling ensembles like BIOMOD. Serra-Diaz J, editor. Diversity and Distributions. 2019; 25: 839–852. https://doi.org/10.1111/ddi.12892
59. Keeley ATH, Beier P, Gagnon JW. Estimating landscape resistance from habitat suitability: effects of data source and nonlinearities. Landscape Ecology. 2016; 31: 2151–2162. https://doi.org/10.1007/s10980-016-0387-5

60. McClure ML, Hansen AJ, Inman RM. Connecting models to movements: testing connectivity model predictions against empirical migration and dispersal data. Landscape Ecology. 2016; 31: 1419–1432. https://doi.org/10.1007/s10980-016-0347-0
61. Jackson CR, Marnewick K, Lindsey PA, Røskaft E, Robertson MP. Evaluating habitat connectivity methodologies: a case study with endangered African wild dogs in South Africa. Landscape Ecology. 2016; 31: 1433–1447. https://doi.org/10.1007/s10980-016-0342-5
62. Zeller KA, Jennings MK, Vickers TW, Ernest HB, Cushman SA, Boyce WM. Are all data types and connectivity models created equal? Validating common connectivity approaches with dispersal data. Bolliger J, editor. Diversity and Distributions. 2018; 24: 868–879. https://doi.org/10.1111/ddi.12742
63. Caro T. Conservation by proxy: Indicator, Umbrella, Keystone, Flagship and other surrogate species. Washington, DC: Island Press; 2010.
64. Kiffner C, Wenner C, Laviolet A, Yeh K, Kioko J. From savannah to farmland: Effects of land-use on mammal communities in the Tarangire-Manyara ecosystem, Tanzania. African Journal of Ecology. 2015; 53: 156–166. https://doi.org/10.1111/aje.12160
65. Kiffner C, Nagar S, Kollmar C, Kioko J. Wildlife species richness and densities in wildlife corridors of Northern Tanzania. Journal for Nature Conservation. 2016; 31: 29–37. https://doi.org/10.1016/j.jnc.2016.02.006
66. Morrison TA, Bolger DT. Wet season range fidelity in a tropical migratory ungulate. Journal of Animal Ecology. 2012; 81: 543–552. https://doi.org/10.1111/j.1365-2656.2011.01941.x PMID: 22256947
67. Borner M. The increasing isolation of Tarangire National Park. Oryx. 1985; 19: 91–96. https://doi.org/10.1017/S0030605300019797
68. Gereta E, Meing'ataki GEO, Mduma S, Wolanski E. The role of wetlands in wildlife migration in the Tarangire ecosystem, Tanzania. Wetlands Ecology and Management. 2005; 12: 285–299. https://doi.org/10.1007/s11273-005-3499-2
69. Kahurananga J, Silkiluwasha F. The migration of zebra and wildebeest between Tarangire National Park and Simanjiro Plains, northern Tanzania, in 1972 and recent trends. African Journal of Ecology. 1997; 35: 179–185. https://doi.org/10.1111/j.1365-2028.1997.071-89071.x
70. Lohay GG, Riggio J, Lobora AL, Kissui BM, Morrison TA. Wildlife movements and landscape connectivity in the Tarangire Ecosystem. In: Kiffner C, Bond ML, Lee DE, editors. Tarangire: Human-Wildlife Coexistence in a Fragmented Ecosystem. Springer; 2022.
71. Prins HHT, Loth PE. Rainfall Patterns as Background to Plant Phenology in Northern Tanzania. Journal of Biogeography. 1988; 15: 451. https://doi.org/10.2307/2845275
72. Msoffe FU, Said MY, Ogutu JO, Kifugo SC, de Leeuw J, van Gardingen P, et al. Spatial correlates of land-use changes in the Maasai- Steppe of Tanzania: Implications for conservation and environmental planning. International Journal of Biodiversity and Conservation. 2011; 3: 280–290. Available: http://www.academicjournals.org/ijbc
73. Caro T, Davenport TRB. Wildlife and wildlife management in Tanzania. Conservation Biology. 2016; 30: 716–723. https://doi.org/10.1111/cobi.12658 PMID: 26681228
74. Kioko J, Zink E, Sawdy M, Kiffner C. Elephant (Loxodonta africana) Demography and Behaviour in the Tarangire-Manyara Ecosystem, Tanzania. South African Journal of Wildlife Research. 2013; 43: 44–51. https://doi.org/10.3957/056.043.0109
75. Kioko J, Herbert V, Mwetta D, Kilango Y, Murphy-Williams M, Kiffner C. Environmental Correlates of African Elephant (Loxodonta africana) Distribution in Manyara Area, Tanzania. Annual Research & Review in Biology. 2014; 5: 147–154. https://doi.org/10.9734/arrb/2015/12470
76. Yamashita T, Gaynor KM, Kioko J, Brashares JS, Kiffner C. Antipredator behaviour of African ungulates around human settlements. African Journal of Ecology. 2018; 56: 528–536. https://doi.org/10.1111/aje.12489
77. Latham J, Cumani R, Rosati I, Bloise M. FAO Global Land Cover SHARE Database Beta-Release Version 1.0. 2014. Available: http://www.glcn.org/downs/prj/glcshare/GLC_SHARE_beta_v1.0_2014.pdf
78. Pekel J-F, Cottam A, Gorelick N, Belward AS. High-resolution mapping of global surface water and its long-term changes. Nature. 2016; 540: 418–422. https://doi.org/10.1038/nature20584 PMID: 27926733
79. Dormann CF, Elith J, Bacher S, Buchmann C, Carl G, Carré G, et al. Collinearity: A review of methods to deal with it and a simulation study evaluating their performance. Ecography. 2013; 36: 027–046. https://doi.org/10.1111/j.1600-0587.2012.07348.x
80. R Core Team. R: A Language and Environment for Statistical Computing. Vienna, Austria: R Foundation for Statistical Computing; 2021. Available: https://www.r-project.org
81. van Proosdij ASJ, Sosef MSM, Wieringa JJ, Raes N. Minimum required number of specimen records to develop accurate species distribution models. Ecography. 2016; 39: 542–552. https://doi.org/10.1111/ecog.01509

82. Barbet-Massin M, Jiguet F, Albert CH, Thuiller W. Selecting pseudo-absences for species distribution models: How, where and how many? Methods in Ecology and Evolution. 2012; 3: 327–338. https://doi.org/10.1111/j.2041-210X.2011.00172.x

83. Pullinger MG, Johnson CJ. Maintaining or restoring connectivity of modified landscapes: Evaluating the least-cost path model with multiple sources of ecological information. Landscape Ecology. 2010; 25: 1547–1560. https://doi.org/10.1007/s10980-010-9526-6

84. Belote RT, Dietz MS, McRae BH, Theobald DM, McClure ML, Hugh Irwin G, et al. Identifying corridors among large protected areas in the United States. PLoS ONE. 2016; 11: e0154223. https://doi.org/10.1371/journal.pone.0154223 PMID: 27104683

85. McRae BH, Dickson BG, Keitt TH, Shah VB. Using circuit theory to model connectivity in ecology, evolution, and conservation. Ecology. 2008; 89: 2712–24. https://doi.org/10.1890/07-1861.1 PMID: 18959309

86. Adriaensen F, Chardon JP, de Blust G, Swinnen E, Villalba S, Gulinck H, et al. The application of "least-cost" modelling as a functional landscape model. Landscape and Urban Planning. 2003; 64: 233–247. https://doi.org/10.1016/S0169-2046(02)00242-6

87. McRae BH, Kavanagh DM. Linkage Mapper Connectivity Analysis Software. Seattle, WA: The Nature Conservancy; 2011.

88. Pinto N, Keitt TH. Beyond the least-cost path: Evaluating corridor redundancy using a graph-theoretic approach. Landscape Ecology. 2009; 24: 253–266. https://doi.org/10.1007/s10980-008-9303-y

89. Sawyer SC, Epps CW, Brashares JS. Placing linkages among fragmented habitats: Do least-cost models reflect how animals use landscapes? Journal of Applied Ecology. 2011; 48: 668–678. https://doi.org/10.1111/j.1365-2664.2011.01970.x

90. Bolger DT, Newmark WD, Morrison TA, Doak DF. The need for integrative approaches to understand and conserve migratory ungulates. Ecology Letters. 2008; 11: 63–77. https://doi.org/10.1111/j.1461-0248.2007.01109.x PMID: 17897327

91. Runge CA, Martin TG, Possingham HP, Willis SG, Fuller RA. Conserving mobile species. Frontiers in Ecology and the Environment. 2014; 12: 395–402. https://doi.org/10.1890/130237

92. Seidler RG, Long RA, Berger J, Bergen S, Beckmann JP. Identifying impediments to long-distance mammal migrations. Conservation Biology. 2015; 29: 99–109. https://doi.org/10.1111/cobi.12376 PMID: 25158993

93. Thuiller W, Broennimann O, Hughes G, Alkemade JRM, Midgley GF, Corsi F. Vulnerability of African mammals to anthropogenic climate change under conservative land transformation assumptions. Global Change Biology. 2006; 12: 424–440. https://doi.org/10.1111/j.1365-2486.2006.01115.x

94. Carroll C, Noss RF. Rewilding in the face of climate change. Conservation Biology. 2021; 35: 155–167. https://doi.org/10.1111/cobi.13531 PMID: 32557877

95. Lawler JJ, Rinnan DS, Michalak JL, Withey JC, Randels CR, Possingham HP. Planning for climate change through additions to a national protected area network: implications for cost and configuration. Philosophical Transactions of the Royal Society B. 2020;375. https://doi.org/10.1098/rstb.2019.0117 PMID: 31983335

96. Bennitt E, Hubel TY, Bartlam-Brooks H, Wilson AM. Possible causes of divergent population trends in sympatric African herbivores. Yue B-S, editor. PLoS ONE. 2019; 14: e0213720. https://doi.org/10.1371/journal.pone.0213720 PMID: 30861044

97. Abrahms B, Sawyer SC, Jordan NR, McNutt JW, Wilson AM, Brashares JS. Does wildlife resource selection accurately inform corridor conservation? Journal of Applied Ecology. 2016; 54: 412–422. https://doi.org/10.1111/1365-2664.12714

98. Gaynor KM, Hojnowski CE, Carter NH, Brashares JS. The influence of human disturbance on wildlife nocturnality. Science. 2018; 360: 1232–1235. https://doi.org/10.1126/science.aar7121 PMID: 29903973

99. Gaynor KM, Branco PS, Long RA, Gonçalves DD, Granli PK, Poole JH. Effects of human settlement and roads on diel activity patterns of elephants (Loxodonta africana). African Journal of Ecology. 2018; 56: 872–881. https://doi.org/10.1111/aje.12552

100. Morrison TA, Link WA, Newmark WD, Foley CAH, Bolger DT. Tarangire revisited: Consequences of declining connectivity in a tropical ungulate population. Biological Conservation. 2016; 197: 53–60. https://doi.org/10.1016/j.biocon.2016.02.034

101. Naidoo R, Kilian JW, du Preez P, Beytell P, Aschenborn O, Taylor RD, et al. Evaluating the effectiveness of local- and regional-scale wildlife corridors using quantitative metrics of functional connectivity. Biological Conservation. 2018; 217: 96–103. https://doi.org/10.1016/j.biocon.2017.10.037

102. Mwalyosi RBB. Ecological evaluation for wildlife corridors and buffer zones for Lake Manyara National Park, Tanzania, and its immediate environment. Biological Conservation. 1991; 57: 171–186. https://doi.org/10.1016/0006-3207(91)90137-X

103. Araújo MB, New M. Ensemble forecasting of species distributions. Trends in Ecology and Evolution. 2007; 22: 42–47. https://doi.org/10.1016/j.tree.2006.09.010 PMID: 17011070

104. Diplock N, Johnston K, Mellon A, Mitchell L, Moore M, Schneider D, et al. Large mammal declines and the incipient loss of mammal-bird mutualisms in an African savanna ecosystem. de Boer WF, editor. PLoS ONE. 2018; 13: e0202536. https://doi.org/10.1371/journal.pone.0202536 PMID: 30153277
